# Morphological and functional alterations in type 2 diabetes pancreata assessed with MRI-based metrics and [^18^F]FP-(+)-DTBZ PET

**DOI:** 10.3389/fendo.2025.1724340

**Published:** 2025-12-18

**Authors:** Seyed Faraz Nejati, Faranak Ebrahimian Sadabad, Rui Ren, Yuan Huang, Jason Bini

**Affiliations:** 1 Positron Emission Tomography (PET)Center, Yale Biomedical Imaging Institute, Department of Radiology and Biomedical Imaging, Yale University, New Haven, CT, United States; 2 Department of Biostatistics, Yale School of Public Health, Yale University, New Haven, CT, United States

**Keywords:** positron emission tomography (PET), magnetic resonance imaging (MRI), pancreas, diabetes, insulin

## Abstract

**Objective:**

To determine if combining PET-derived beta-cell mass (BCM) estimates with MRI-based morphology metrics improves the prediction of beta-cell functional mass in type 2 diabetes (T2D).

**Methods:**

We performed a retrospective analysis of 40 participants—19 T2D individuals, 16 healthy obese volunteers (HOVs), and five prediabetes individuals—who underwent [^18^F]FP-(+)-DTBZ PET to quantify vesicular monoamine transporter type 2 (VMAT2) density [standardized uptake value ratio (SUVR-1)], T1-weighted MRI for 3D morphology metric analysis, and an arginine stimulation test to measure acute (AIRarg) and maximum (AIRargMAX) insulin responses. Least Absolute Shrinkage and Selection Operator (LASSO) regression models identified the optimal combination of positron emission tomography (PET), MRI, and clinical variables to predict beta-cell function for the whole pancreas and its subregions.

**Results:**

Compared to HOVs, individuals with T2D exhibited significantly reduced AIRarg and AIRargMAX. Only the pancreas body volume was significantly smaller in the T2D cohort. For the whole pancreas, a model including PET-derived SUVR-1 and a subset of clinical covariates best predicted acute beta-cell function (AIRarg). However, predicting maximum functional reserve (AIRargMAX) required the addition of MRI-based morphology metrics in combination with SUVR-1 and a subset of clinical covariates.

**Conclusion:**

We combined PET imaging of BCM and MRI morphology metrics with a robust machine learning-based variable selection method to extract useful PET- and MRI-based metrics for predicting acute and maximum insulin responses. This synergistic approach offers a novel combination of biomarkers for staging disease and evaluating therapeutic interventions.

## Introduction

Type 2 diabetes (T2D) is characterized by chronic insulin resistance, increased β-cell workload to failure, and eventual decline in β-cell function and mass in the pancreas (1). Several imaging studies have demonstrated pancreatic volume loss ranging from 13% to 33% (2–5). Along with pancreatic volume loss, autopsy studies have demonstrated loss of 40%–65% of beta-cell mass (BCM) in individuals with T2D (6, 7). Only 1%–3% of pancreas volume consists of islet mass; therefore, understanding the mechanisms of pancreatic volume loss in both the endocrine and exocrine pancreas in T2D is important.

Several biomarkers are currently used to assess endocrine and exocrine function in the pancreas (8–11). Endocrine pancreas function can be assessed using peripheral blood measurements such as hemoglobin A1c (HbA1c), insulin, C-peptide, and proinsulin, as well as the ratio of proinsulin to C-peptide (PI:C ratio) (8–10). More rigorous tests of functional BCM can be performed, such as the arginine stimulation test (AST) (12, 13). Intravenous arginine is an ideal β- and α-cell agonist that allows simultaneous examination of insulin, C-peptide, and glucagon responses (12–14). Exocrine pancreas secretory enzymes such as amylase, lipase, and trypsinogen have been proposed as serological biomarkers with relationships to pancreatic volume loss (11). Although recent studies have demonstrated the clinical utility of serum biomarkers, such as PI:C ratio, to assess treatment response (10), further understanding of the relationship between serum biomarkers and both endocrine and exocrine pancreas structure and function is necessary.

To understand the fate of beta-cells during clinical diabetes, measurement of BCM *in vivo* is largely performed using positron emission tomography (PET) imaging with radioligands that bind primarily to receptor targets on beta-cells. There are several targets that are currently being pursued, including vesicular monoamine transporter type 2 (VMAT2), dopamine receptors, and GLP-1 receptors, with PET (3, 15–18) or single-photon emission computed tomography (SPECT) (19). VMAT2, primarily expressed within beta-cells, is a transmembrane protein responsible for sequestering insulin and dopamine into insulin-secretory granules to regulate insulin secretion (20). [^18^F]Fluoropropyl-dihydrotetrabenazine ([^18^F]FP-(+)-DTBZ) is a radioligand that binds to VMAT2 and has been shown to highly correlate with BCM (3, 21–27). Initial human studies using [^18^F]FP-(+)-DTBZ to measure BCM were performed in patients with type 1 diabetes (T1D) (22, 24); however, recently, this method was extended to patients with T2D (3). In the T2D study, correlations of VMAT2 density, a biomarker of BCM, correlated with years of T2D diagnosis, glycemic control, and beta-cell functional measures, suggesting that PET was able to quantify changes in BCM (3). Magnetic resonance imaging (MRI) in the same subjects revealed a pancreatic volume decrease of ~13% in T2D, compared to healthy obese volunteers (HOVs), suggesting loss of both endocrine BCM and exocrine volume.

Cross-sectional and longitudinal studies in patients with T1D or T2D have examined the role of pancreas volume or pancreatic volume index (PVI), volume normalized to body weight (28–31). More recently, the identification of pancreas morphology metrics, for example, surface area, long and short axis lengths, and ratio of longest to shortest axes, has revealed more complex changes in pancreas structure beyond pancreas volume and PVI in longitudinal studies of patients with T1D (30, 31). Combining PVI and pancreas morphology metric classification of individuals with T1D versus healthy controls improved compared to using only PVI (30, 31). Similar MRI-based morphology metrics have been proposed in T2D (2, 4, 32). Beyond structural MRI, additional methods taking advantage of beta-cell metabolism, such as manganese-enhanced magnetic resonance imaging, have been explored (33).

To our knowledge, no one has combined PET BCM measurements with pancreas morphology metrics, beyond pancreas volume or PVI, as was performed previously (3). In this retrospective study, we re-examined PET and MRI data with the addition of new pancreas morphology metrics to reveal important endocrine and exocrine pancreas features that may predict BCM and function in T2D. This was performed by reporting pancreas MRI morphology metrics in healthy obese volunteers (HOVs) and patients with T2D and combining MRI and PET metrics in a logistic regression that predicted functional BCM. Exploratory group difference analyses and linear correlations also revealed that both PET and MRI morphology metrics can predict aspects of beta-cell mass and function in T2D.

## Materials and methods

This is a retrospective exploratory analysis of the previously published [^18^F]FP-(+)-DTBZ PET imaging study (3). The study was approved by the Yale University Human Investigation Committee and the Yale-New Haven Hospital Radiation Safety Committee and followed the federal guidelines and regulations of the USA for the protection of human research subjects contained in Title 45 Part 46 of the Code of Federal Regulations (45 CFR 46). All participants provided signed written informed consent.

Briefly, the original study included 40 participants: 16 HOVs, five individuals with prediabetes, and 19 individuals with T2D. As described previously (3), age- and body mass index (BMI) -matched HOVs had no history of type 1 or type 2 diabetes or were diagnosed with prediabetes, with the following results: HbA1c < 39 mmol/mol (5.7%), fasting blood glucose (FBG) <5.6 mmol/L, and 75-g oral glucose tolerance test (OGTT) with 2-h post-challenge glucose <7.8 mmol/L. All subjects underwent pancreas [^18^F]FP-(+)-DTBZ PET, pancreas MRI acquisition, and an arginine stimulation test (AST). AST provided two outcome measures, acute insulin response to arginine (AIRarg; serum C-peptide from 0 to 5 min) and maximum insulin response to arginine (AIRargMAX; serum C-peptide from 55 to 65 min) (12, 13). As described previously (3), the PET study consisted of a 2-h scan (SCAN-A) and a 1.5-h scan (SCAN-B), with a 30-min break between the two scans. The frame timing of PET data was 6 × 30 s, 3 × 1 min, 2 × 2 min, and 22 × 5 min (SCAN-A) and 18 × 5 min (SCAN-B). PET images were reconstructed using the ordered subset expectation maximization algorithm with point spread function correction using time-of-flight measurements. Further details regarding PET and MR acquisition, AST protocol, and quantitative PET analyses can be found in the previous manuscript (3). To avoid partial volume effects, pancreas and spleen regions of interest (ROIs) were thinned using the “classical thinning algorithm”, as described in the previous manuscript (3). Here, the same quantitative outcome measures from those previously defined ROIs were used.

For the current goal of examining the effectiveness of PET and MR imaging metrics to predict BCM, three outcomes were included: AIRarg (functional beta-cell mass), AIRargMAX (functional and not fully functional beta-cell mass), and the ratio of acute to maximum insulin response to arginine (acute:MAX), reflective of the ratio of functional to functional and not fully functional beta-cell mass. AIRarg reflects beta-cells that immediately release C-peptide during arginine stimulation (<5 min), whereas AIRargMAX is reflective of maximal C-peptide release in response to >60 min of arginine stimulation. Therefore, AIRargMAX is hypothesized to reflect a population of cells that require prolonged stimulation of arginine to release C-peptide and may not respond in normal physiological conditions but have a functional reserve capacity to release C-peptide and insulin (i.e., not fully functional beta-cell mass). Additional clinical measures included age, gender, weight, BMI, HbA1c, years of diabetes, and diagnosis (e.g., HOVs, prediabetes, or T2D).

PET surrogate outcome measures of BCM include the non-displaceable binding potential (*BP*_ND_) (34) and regional standardized uptake value ratio (SUVR-1) computed from the time window (180–240 min) as the ratio of the standardized uptake value (SUV) from two different regions (from a target region and a reference region) using the spleen as a reference region to account for non-specific radiotracer uptake and where subtraction of 1 accounts for non-specific radioligand uptake (e.g., ratio of pancreas non-specific/spleen non-specific) (3). *BP*_ND_ × pancreas volume and SUVR-1 × pancreas volume can also be calculated to account for organ volume loss and are reflective of *aggregate* pancreas BCM.

For the current study, manual pancreas segmentation of the whole pancreas was performed on a T1-weighted abdominal MRI (3). The researcher performing the pancreas segmentation was blinded to the diagnosis of each participant. Following previous methods (30), whole pancreas ROIs were drawn in the axial plane using the Medical Image Processing, Analysis, and Visualization (MIPAV) software, Center for Information Technology, National Institutes of Health, version 11.0.7-2023-06-22 (https://mipav.cit.nih.gov). ROIs were also subdivided into pancreas head, body, and tail. For each subject, the stack of axial pancreas whole, head, body, and tail ROIs was converted to its respective volumes of interest and subsequently to a three-dimensional (3D) binary mask of the pancreas in MIPAV. This 3D binary mask was then used as input into “regionprops3” (MATLAB, version R2023a, The MathWorks, https://www.mathworks.com/products/matlab) to calculate the MRI morphology metrics of the 3D pancreas mask. MRI morphology metric outputs from “regionprops3” include the following: 1) “BoundingBox”, the smallest cuboid containing the pancreas with lengths “BoundingBox1”, “BoundingBox2”, “BoundingBox3”, and “BoundingBoxVolume”; 2) “Centroid”, coordinates of the center of mass of the pancreas (centroid1, centroid2, and centroid3); 3) EquivDiameter, diameter of a sphere with the same volume as the pancreas (e.g., larger EquivDiameter and larger pancreas volume); 4) Extent, ratio of voxels in the pancreas to voxels in the total bounding box; 5) PrincipalAxisLength, length in voxels of the major axes of the ellipsoid that have the same normalized second central moments as the pancreas (PrincipalAxisLength1, PrincipalAxisLength2, and PrincipalAxisLength3); 6) ConvexVolume, number of voxels in the smallest convex polygon that contains the pancreas; 7) Solidity, proportion of voxels in the convex volume that are also in the pancreas; 8) pancreas surface area; and 9) pancreas volume. All metrics were calculated separately for the whole pancreas, and pancreas head, body, and tail.

### Statistical analysis

**Figure 1:**
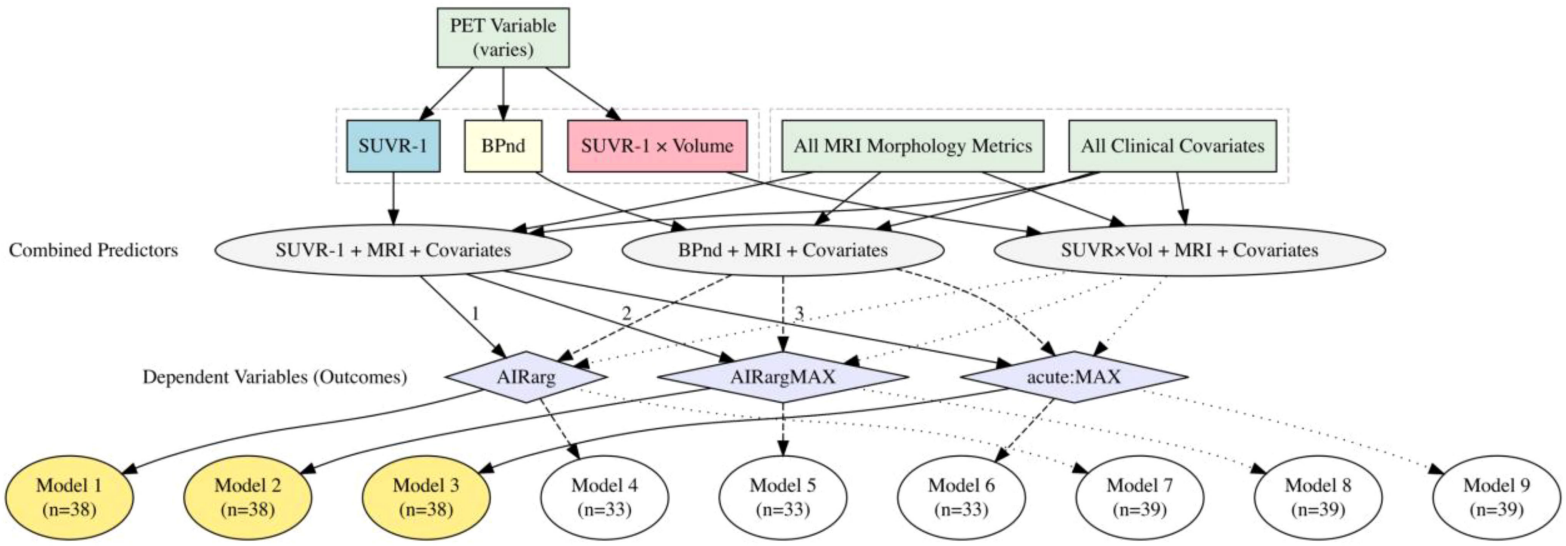
Predictors and outcome composition in full linear regression models. SUVR-1 (blue box) was the primary PET outcome metric used to predict outcomes (purple diamonds). Each model contained combined predictors (gray ovals) consisting of a PET variable, all MRI morphology metrics, and all clinical covariates (green rectangles). Primary outcome models used SUVR-1, all MRI morphology metrics, and all clinical covariates to predict functional BCM outcomes (Models 1–3, yellow circles). Exploratory analyses used *BP*_ND_ or SUVR-1 × Volume with all MRI morphology metrics and all clinical covariates (Models 4–9). SUVR-1, standardized uptake value ratio; BCM, beta-cell mass.

We performed one-way ANOVA for each functional BCM outcome (AIRarg, AIRargMAX, and acute:MAX), and when appropriate, we compared group differences with an unpaired t-test with Welch’s correction. Due to the small sample size of the prediabetes group, all analyses of this cohort were exploratory in nature. We also aimed to investigate the relationships between functional BCM outcomes (AIRarg, AIRargMAX, and acute:MAX) and various predictors [PET variables, MRI morphology metrics, and clinical covariates (e.g., age and BMI)] (**Figure 1**). For each pancreas ROI delineation (whole, head, body, or tail), we constructed three models, each incorporating one PET variable, all MRI morphology metrics, and all clinical covariates for a specific functional BCM outcome. Previously (3), SUVR-1 was the primary outcome variable; therefore, we used that as our primary PET outcome measure (**Figure 1**, yellow circles). We examined additional models as exploratory analyses, using alternate PET outcomes (*BP*_ND_) or pancreas aggregate binding measures (SUVR-1 × Volume) (**Figure 1**, white circles). Differences in sample sizes in each model arose from removing cases with missing data in various combinations of these variables.

### Predictive modeling with variable selection

The primary objective was to determine whether a subset of variables could achieve prediction accuracy comparable to, or better than, a model using all predictors. To identify such a subset, we applied LASSO regression with a bootstrap-based variable-selection procedure to obtain more stable feature selection. LASSO regression is widely used in high-dimensional settings because it shrinks coefficients and selects variables with non-zero estimates, indicating potential relevance to the outcome (33). Given the relatively small sample size and the large number of predictors, we implemented a nested cross-validation with a bootstrap resampling strategy to improve selection stability (**Supplementary Figure 1**). Specifically, we split the data into 10 outer folds. Within each outer training fold, we generated B = 500 bootstrap samples. For each bootstrap replicate, we used an inner 10-fold cross-validation procedure to select the optimal tuning parameter, after which we applied LASSO regression, and we recorded the selected variables. For each variable, we calculated its overall selection frequency as f = C/B, where C is the number of times the variable was selected across all outer training folds and bootstrap iterations. Variables with f > 0.5 were retained for the construction of the final reduced model (34). To evaluate prediction performance, we trained a linear model on each outer training fold using only the final selected variables and computed the squared prediction error ( $y-\hat{y}$)^2^ for all observations in the corresponding outer test fold. Averaging these errors yielded the cross-validated mean squared error (MSE). We then compared the MSE of the full model (including all predictors) with that of the reduced model (including only variables with f > 0.5).

## Results

**Figure 2:**
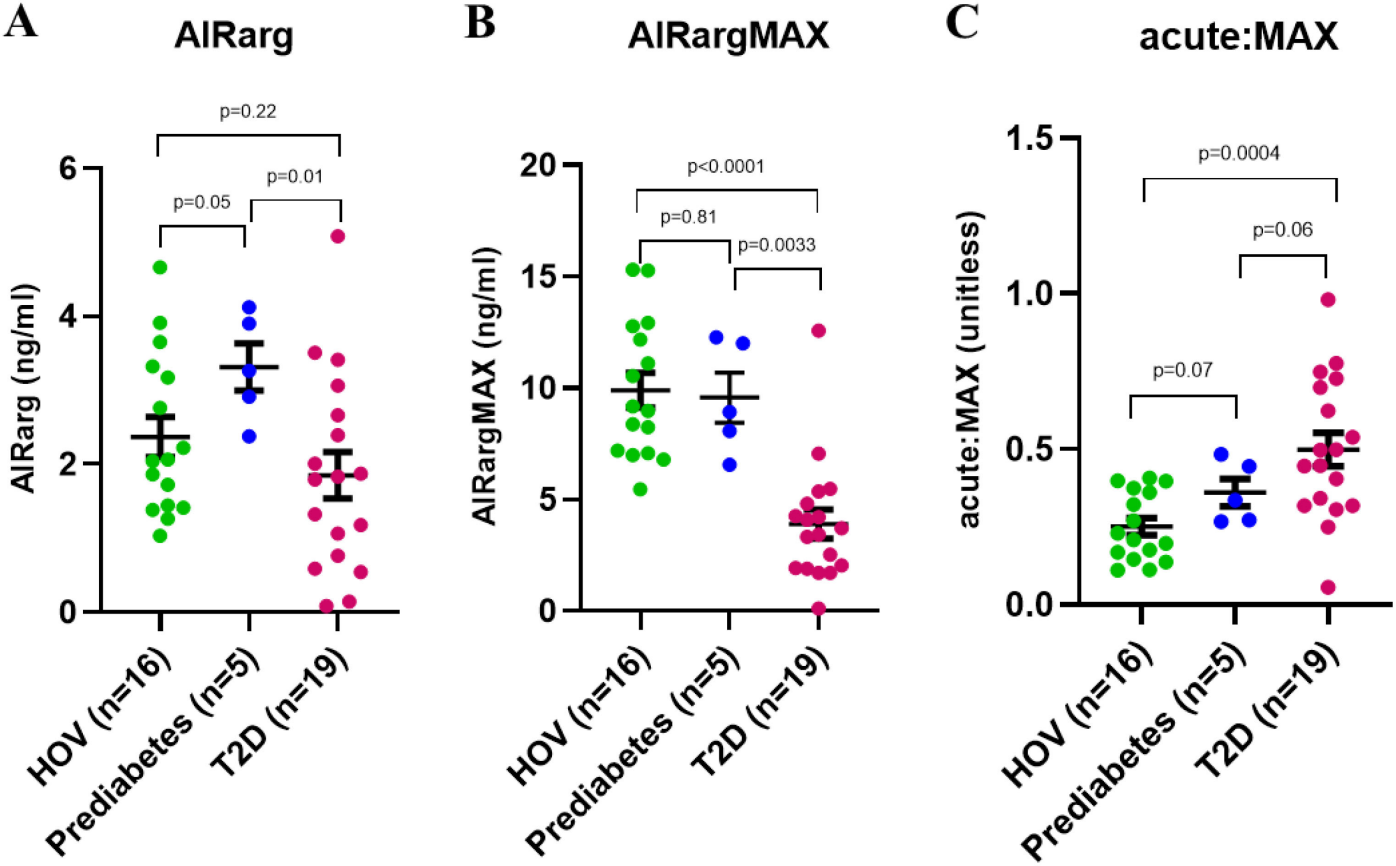
Group comparisons of functional beta-cell mass outcomes. **(A)** Acute insulin response to arginine stimulation (AIRarg). **(B)** Maximum insulin response to arginine (AIRargMAX). **(C)** Ratio of acute to maximum response to arginine (acute:MAX). All data presented as mean ± SEM.

One-way ANOVA demonstrated significant differences in the group means for each of the functional BCM outcomes AIRarg (p = 0.04), AIRargMAX (p < 0.0001), and acute:MAX (p = 0.002); therefore, we examined group differences of the respective outcome measures between the HOV, prediabetes, and T2D groups (**Figure 2**).

AIRarg was higher in those who had prediabetes (mean ± SEM: 3.3 ± 0.3 ng/mL) than in the HOV group (2.4 ± 0.3 ng/mL) but was significantly lower than that in both groups in the patients with T2D (1.9 ± 0.3 ng/mL, p = 0.01) (**Figure 2A**). AIRargMAX was similar in the HOV (9.9 ± 0.8 ng/mL) and prediabetes (9.6 ± 1.1 ng/mL) groups, while in patients with T2D, it was significantly lower (3.9 ± 0.7 ng/mL, p < 0.0001) (**Figure 2B**). For acute:MAX, the values were in rank order, from low to high, between groups HOV (0.25 ± 0.03 unitless) < prediabetes (0.36 ± 0.04 unitless) < T2D (0.50 ± 0.05 unitless); however, only the difference between HOV and T2D was significant (p = 0.0004) (**Figure 2C**).

**Figure 3:**
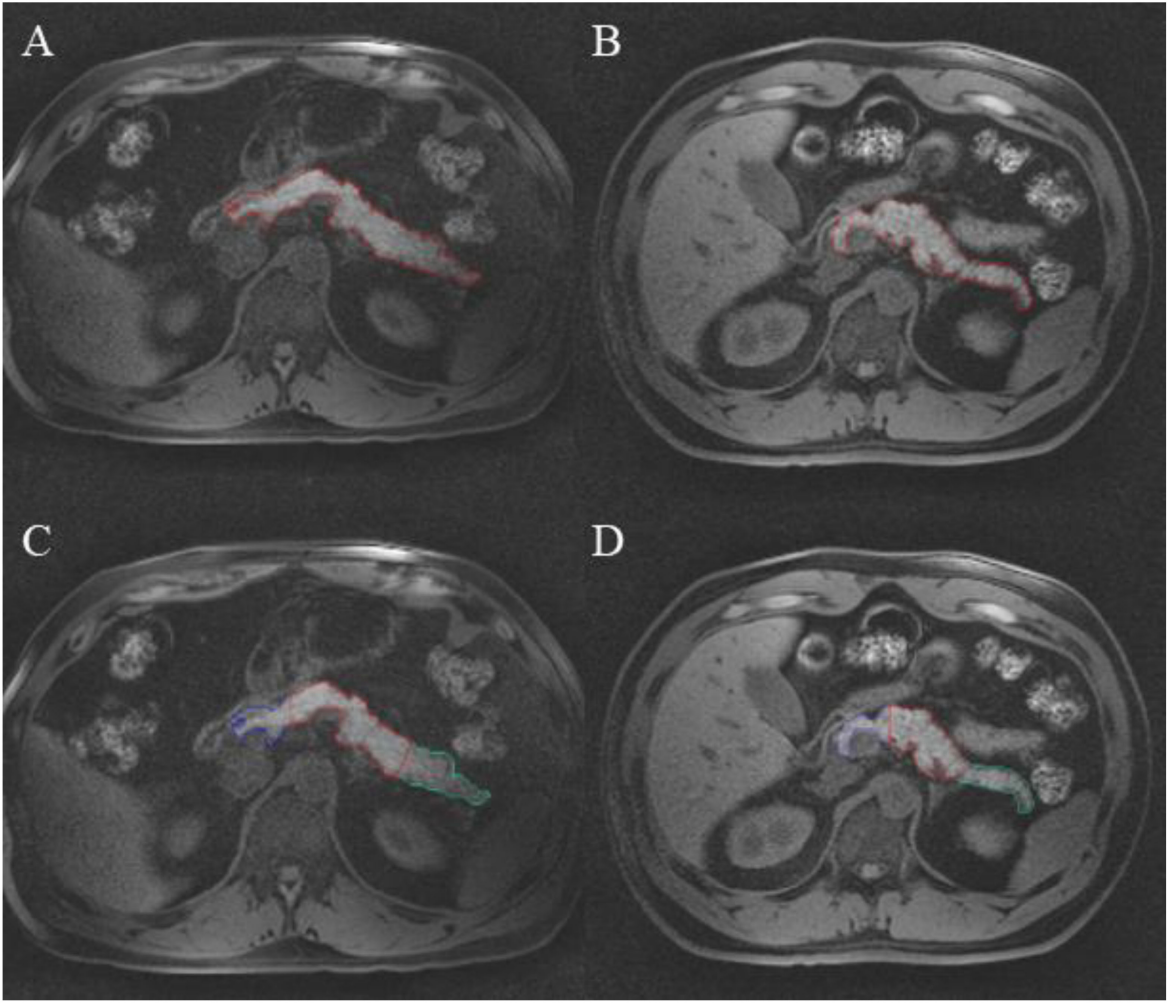
Representative axial pancreas MR images of whole pancreas region of interest in a **(A)** healthy obese volunteer and **(B)** individual with type 2 diabetes. Subdivision of pancreas regions of interest for head (blue), body (red), and tail (green) in **(C)** a healthy obese volunteer and **(D)** an individual with T2D. T2D, type 2 diabetes.

Representative whole pancreas axial slices of the HOV (**Figure 3A**) and T2D (**Figure 3B**) groups were visualized via MRI. Pancreas ROIs in both the HOV (**Figure 3C**) and T2D (**Figure 3D**) groups were subdivided into pancreas head (blue outline), body (red outline), and tail (green outline) ROIs.

**Figure 4:**
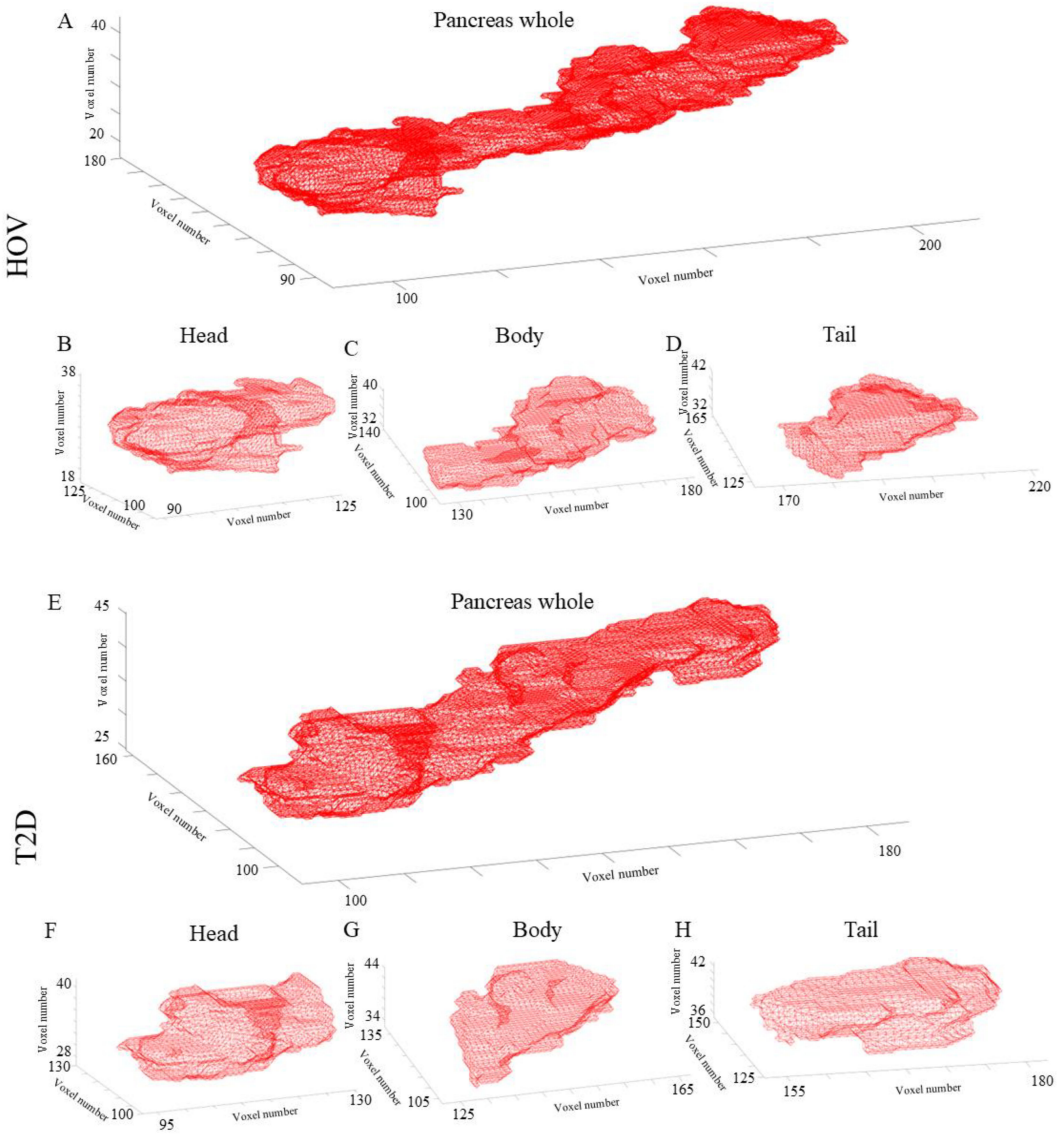
Representative 3D pancreas masks in a healthy obese volunteer: **(A)** whole pancreas and pancreas **(B)** head, **(C)** body, and **(D)** tail. An individual with type 2 diabetes: **(E)** whole pancreas and pancreas **(F)** head, **(G)** body, and **(H)** tail. All axes are voxel numbers. For example, in panel **(A)**, the healthy obese volunteer, the whole pancreas would be roughly bounded by a rectangle of size 100 × 90 × 20 voxels.

A representative stack of axial pancreas whole, head, body, and tail ROIs from an HOV and an individual with T2D converted to their respective 3D binary masks (**Figure 4**).

**Figure 5:**
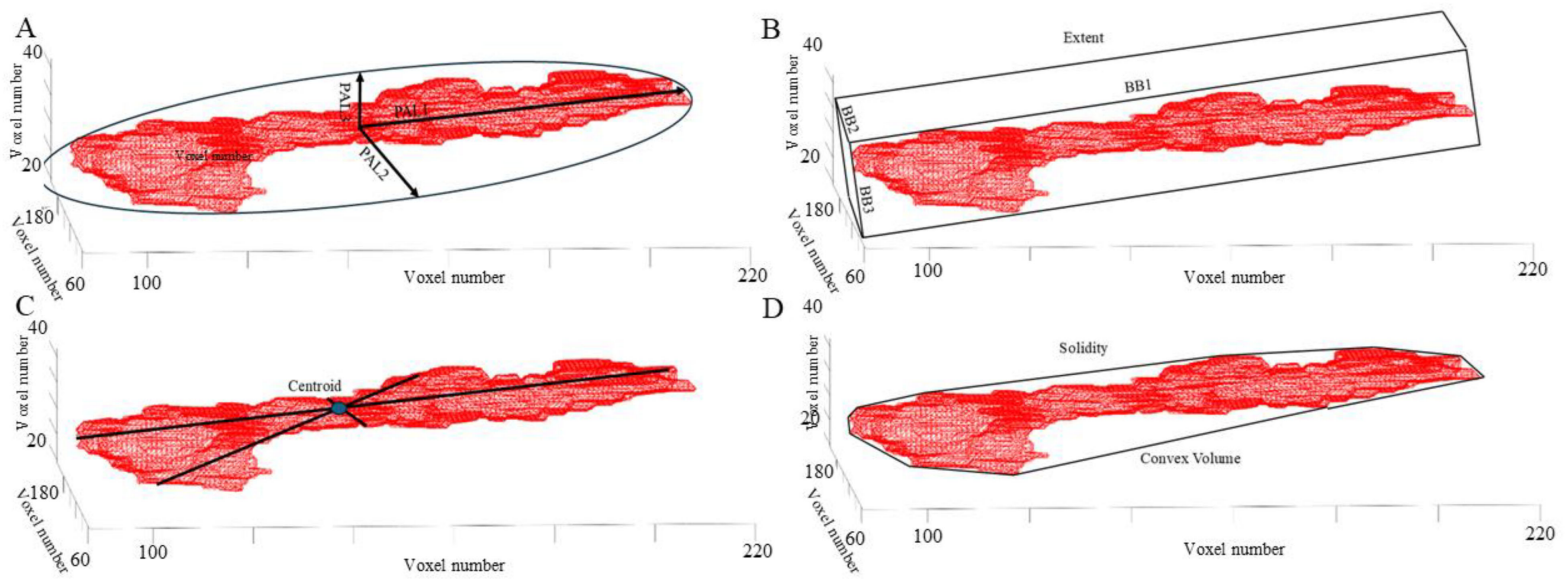
MRI morphology metric outputs from “regionprops3”. **(A)** Principal axis length (PAL) of the smallest ellipsoid encapsulating the pancreas. **(B)** Smallest bounding box (BB) surrounding the pancreas and Extent, the ratio of voxels in the pancreas to voxels in the bounding box. **(C)** Coordinates (x, y, z) of the center of mass of the pancreas (Centroid). **(D)** Smallest Convex Volume surrounding the pancreas and Solidity, the proportion of voxels in the convex volume that are also in the pancreas region.

MRI morphology metric outcomes in a HOV from “regionprops” are presented to demonstrate their relationship to pancreas morphology (**Figure 5**).

No group differences were seen in any pancreas ROI using SUVR-1, as previously reported (**Supplementary Figure 2**) (3). The pancreas body volume was the only ROI that was significantly different between HOVs (mean ± SEM; 39.1 ± 3.4 mL) and individuals with T2D (28.8 ± 3.0 mL, p = 0.01) (**Supplementary Figure 2**).

**Figure 6:**
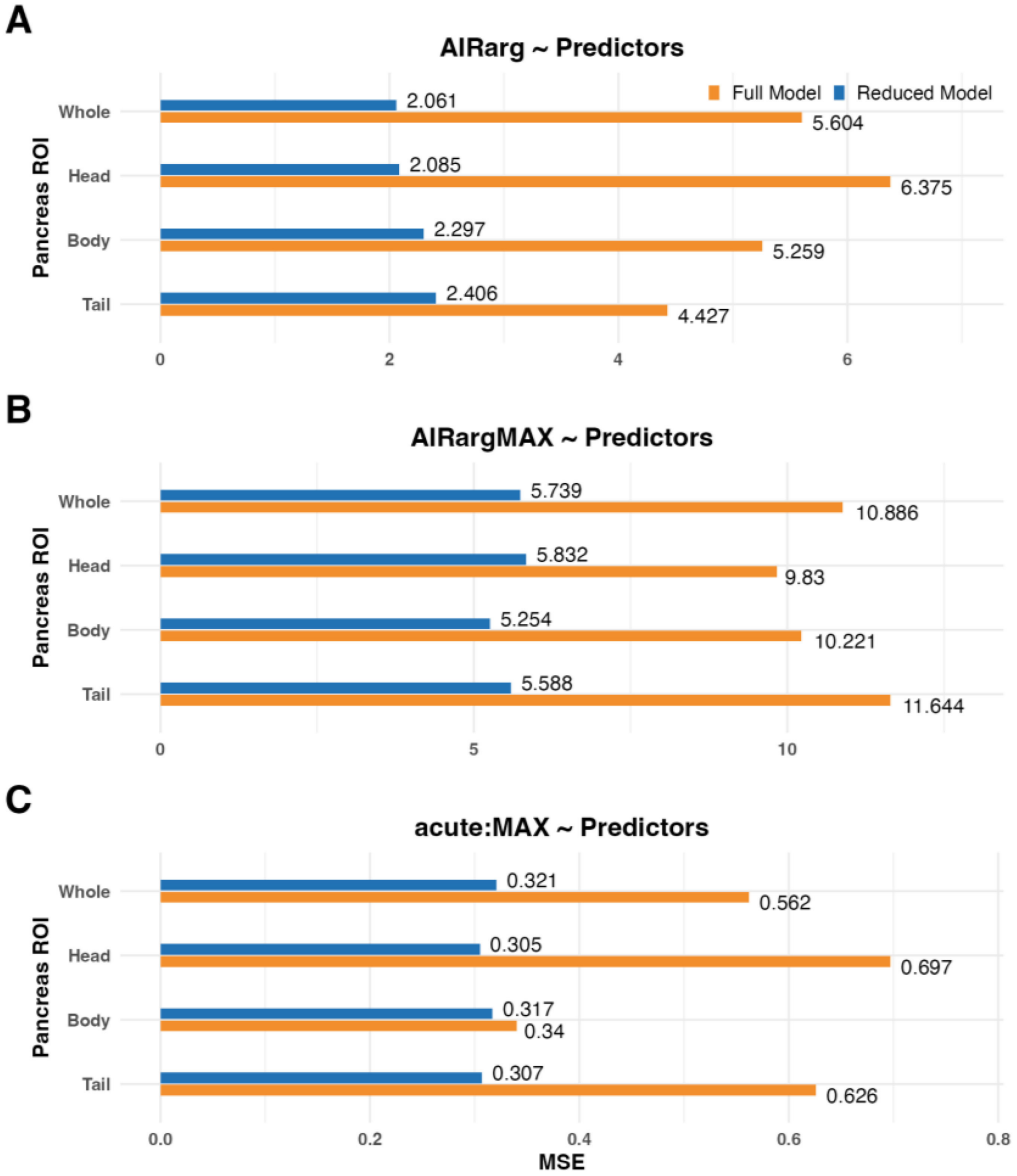
Comparison of cross-validated mean squared error of full (orange bars) and reduced (blue bars) models used to predict functional β-cell mass outcomes: **(A)** AIRarg, **(B)** AIRargMAX, and **(C)** acute:MAX across pancreas and subregions. AIRarg, acute insulin response to arginine; AIRargMAX, maximum insulin response to arginine; acute:MAX, the ratio of acute to maximum insulin response to arginine.

Regression models were analyzed to investigate the relationship between functional beta-cell mass outcomes: AIRarg, AIRargMAX, and acute:MAX, and predictive variables, including the primary PET metric (SUVR-1), MRI morphology metrics, and clinical covariates for whole pancreas and subregions of the pancreas, including head, body, and tail (**Table 1****, ****Supplementary Table 1**). For all linear models and regions, the reduced linear model had lower MSE (**Figure 6****, ****Supplementary Tables 1–4**), indicating that the functional BCM outcomes could be better predicted by a specific subset of PET, MRI, and/or clinical covariates.

**Table 1:** Reduced models for predicting primary functional beta-cell mass outcomes (AIRarg, AIRargMAX, and acute:MAX) with the primary PET outcome measure (SUVR-1), MRI morphology metrics, and clinical covariates.

PET outcome measure	Pancreas ROI	
Linear models to predict acute insulin response to arginine (AIRarg)		
SUVR-1	Whole	AIRarg ~ SUVR-1 [0.615] + age [0.784] + years.of.diabetes [0.771]
	Head	AIRarg ~ SUVR-1 [0.639] + Centroid1 [0.551] + age [0.781] + years.of.diabetes [0.774]
	Body	AIRarg ~ SUVR-1 [0.712] + Centroid1 [0.504] + Centroid2 [0.558] + PrincipalAxisLength3 [0.690] + Solidity [0.612] + age [0.775] + BMI [0.521] + years.of.diabetes [0.774]
	Tail	AIRarg ~ SUVR-1 [0.585] + Centroid2 [0.500] + Centroid3 [0.546] + BoundingBox1 [0.650] + PrincipalAxisLength3 [0.543] + age [0.857] + BMI [0.599] + HbA1c [0.504] + years.of.diabetes [0.802]
Linear models to predict maximum insulin response to arginine (AIRargMAX)		
SUVR-1	Whole	AIRargMAX ~ SUVR-1 [0.788] + Centroid1 [0.543] + Centroid2 [0.807] + Centroid3 [0.851] + PrincipalAxisLength3 [0.581] + age [0.593] + BMI [0.525] + HbA1c [0.966] + years.of.diabetes [0.800]
	Head	AIRargMAX ~ SUVR-1 [0.699] + Centroid1 [0.584] + Centroid2 [0.834] + Centroid3 [0.710] + PrincipalAxisLength1 [0.507] + PrincipalAxisLength2 [0.854] + PrincipalAxisLength3 [0.618] + age [0.615] + HbA1c [0.873] + years.of.diabetes [0.871]
	Body	AIRargMAX ~ SUVR-1 [0.719] + Centroid1 [0.608] + Centroid2 [0.849] + Centroid3 [0.841] + PrincipalAxisLength1 [0.515] + PrincipalAxisLength2 [0.557] + PrincipalAxisLength3 [0.805] + age [0.686] + weight [0.511] + HbA1c [0.950] + years.of.diabetes [0.722]
	Tail	AIRargMAX ~ SUVR-1 [0.680] + Centroid1 [0.678] + Centroid2 [0.634] + Centroid3 [0.749] + PrincipalAxisLength3 [0.502] + age [0.561] + HbA1c [0.949] + years.of.diabetes [0.813]
Linear models to predict ratio of acute to maximum insulin response to arginine (acute:MAX)		
SUVR-1	Whole	acute:MAX ~ SUVR-1 [0.622] + Centroid1 [0.727] + Centroid2 [0.551] + Centroid3 [0.573] + BoundingBox1 [0.572] + BoundingBox3 [0.662] + PrincipalAxisLength1 [0.582] + PrincipalAxisLength2 [0.512] + PrincipalAxisLength3 [0.690] + age [0.705] + BMI [0.509] + HbA1c [0.977] + years.of.diabetes [0.635]
	Head	acute:MAX ~ SUVR-1 [0.588] + PrincipalAxisLength1 [0.536] + PrincipalAxisLength2 [0.625] + PrincipalAxisLength3 [0.607] + age [0.628] + HbA1c [0.967] + years.of.diabetes [0.558]
	Body	acute:MAX ~ SUVR-1 [0.717] + Centroid1 [0.699] + Centroid2 [0.642] + Centroid3 [0.636] + BoundingBox1 [0.648] + BoundingBox2 [0.532] + BoundingBox3 [0.588] + Extent [0.612] + PrincipalAxisLength2 [0.721] + Solidity [0.560] + age [0.730] + weight [0.560] + HbA1c [0.986] + years.of.diabetes [0.684]
	Tail	acute:MAX ~ SUVR-1 [0.510] + Centroid1 [0.719] + Centroid2 [0.560] + Centroid3 [0.516] + PrincipalAxisLength2 [0.614] + age [0.684] + HbA1c [0.970] + years.of.diabetes [0.587]

Values in brackets [] denote the selection frequencies of variables retained in each reduced model.

SUVR-1, standardized uptake value ratio; ROI, region of interest; HbA1c, hemoglobin A1c.

In the reduced models for predicting AIRarg, SUVR-1 was included for the whole pancreas and every subregion (**Table 1****, ****Supplementary Table 1**). No MRI morphology metrics were included in the reduced models for the whole pancreas. In the pancreas head, only centroid 1 was included. For the pancreas body, centroid 1 and 2, principal axis length 3, and solidity were included; in the tail, only centroid 2 and 3, principal axis 3, and bounding box 1 were included in the reduced model.

For reduced models predicting AIRargMAX, SUVR-1 was again included for the whole pancreas and each subregion. For MRI morphology metrics, the whole pancreas was comprised of centroids 1–3 and principal axis length 3. The head and body included centroids 1–3 and principal axis lengths 1–3. For the tail, centroids 1–3 and only principal axis 3 were included.

In the final model, to predict acute:MAX, SUVR-1 was included in all reduced models. The MRI morphology metrics included several variations, dependent on pancreas region, of the following parameters: centroids 1–3, bounding boxes 1–3, principal axes 1–3, extent, and solidity.

Exploratory reduced models, using alternate PET outcomes (*BP*_ND_) or pancreas volume aggregate binding measures (SUVR-1 × Volume or *BP*_ND_ × Volume), showed similar patterns of reduced MSE and combinations of PET and MRI morphology metrics (**Supplementary Tables 2–4**).

**Figure 7:**
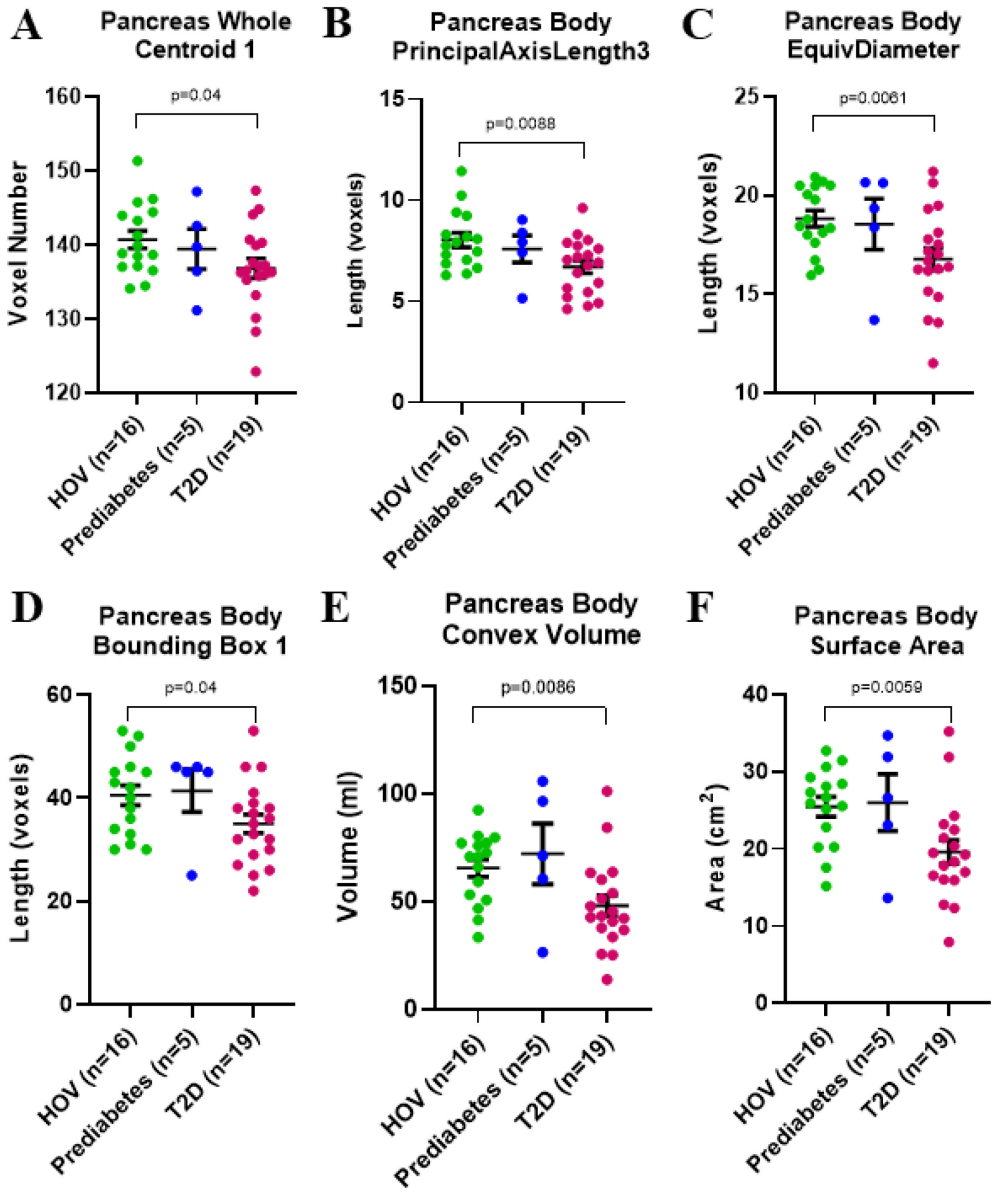
Exploratory group comparisons of pancreas MRI morphology metrics between healthy obese volunteers and individuals with T2D in whole pancreas for **(A)** Centroid 1, and in pancreas body for **(B)** PrincipalAxisLength3, **(C)** EquivDiameter, **(D)** Bounding Box 1, **(E)** Convex Volume, and **(F)** Surface Area. All data presented as mean ± SEM. T2D, type 2 diabetes.

Exploratory analyses examining group differences (mean ± SEM) of pancreas MRI morphology metrics revealed significant differences in whole pancreas centroid 1 (HOV: 140.7 ± 1.2 voxel number, T2D: 136.9 ± 1.3 voxel number; p = 0.04), pancreas body principal axis length 3 (HOV: 8.0 ± 0.5 voxels, T2D: 6.7 ± 0.3 voxels; p = 0.0088), pancreas body EquivDiameter (HOV: 18.8 ± 0.7 voxels, T2D: 16.8 ± 0.6; voxels p = 0.0061), pancreas body bounding box 1 (HOV: 40.5 ± 2.2 voxels, T2D: 35 ± 1.9 voxels; p = 0.04), pancreas body convex volume (HOV: 65.6 ± 5.6 mL, T2D: 48.1 ± 5.7 mL; p = 0.0086), and pancreas body surface area (HOV: 25.5 ± 1.8 cm^2^, T2D: 19.6 ± 1.6 cm^2^; p = 0.0059) (**Figure 7**).

**Figure 8:**
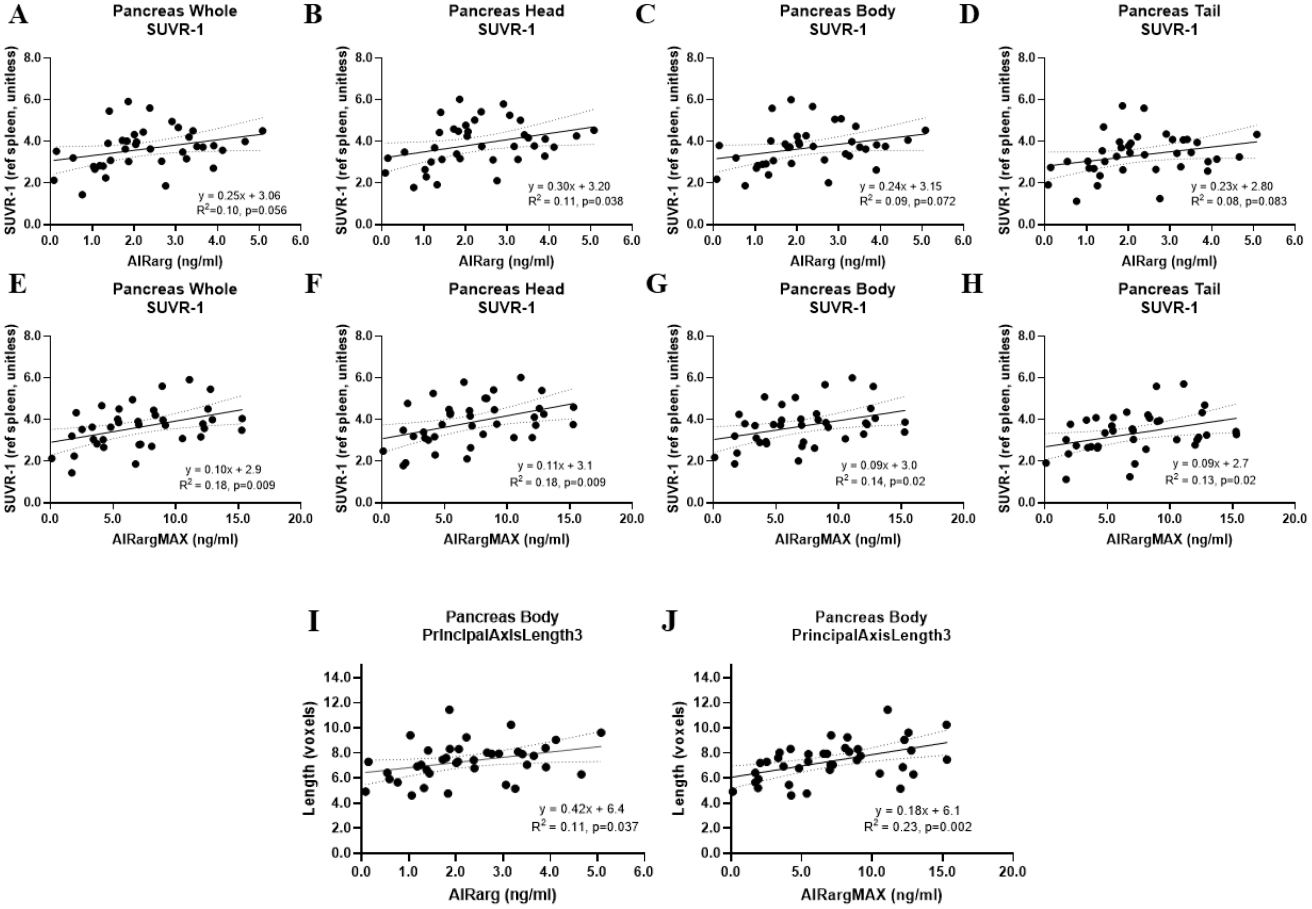
Exploratory correlations between functional beta-cell mass outcome and imaging metrics. Correlations of AIRarg with SUVR-1 in all pancreas regions: **(A)** whole, **(B)** head, **(C)** body, and **(D)** tail. Correlations of AIRargMAX with SUVR-1 in all pancreas regions: **(E)** whole, **(F)** head, **(G)** body, and **(H)** tail. Correlations of Pancreas Body PrincipalAxisLength3 with **(I)** AIRarg and **(J)** AIRargMAX. Dotted lines, 95% confidence interval for line of regression. AIRarg, acute insulin response to arginine; SUVR-1, standardized uptake value ratio; AIRargMAX, maximum insulin response to arginine.

Exploratory correlations between AIRarg, AIRargMAX, acute:MAX, and single PET and MRI morphology metrics are displayed to compare differences and similarities of PET and MRI morphology metrics to functional BCM outcome measures (**Figure 8**).

AIRarg correlated with both pancreas head SUVR-1 (R^2^ = 0.11, p = 0.036) and pancreas body Principal axis length 3 (PAL3) (R^2^ = 0.11, p = 0.037). As previously reported (3), AIRargMAX was significantly correlated with SUVR-1 in all pancreas regions: whole (R^2^ = 0.18, p = 0.009), head (R^2^ = 0.18, p = 0.009), body (R^2^ = 0.14, p = 0.02), and tail (R^2^ = 0.13, p = 0.02). However, AIRargMAX was also significantly correlated with the MRI morphology metric pancreas body principal axis length 3 (R^2^ = 0.23, p = 0.002). No standalone imaging metrics were correlated with acute:MAX.

## Discussion

We performed a retrospective analysis of PET and MRI pancreas imaging data with new analyses of MRI morphology metrics to determine which combination of imaging-based metrics best predicts beta-cell mass and function in patients with T2D.

Functional beta-cell mass assessments showed significant differences between HOV and patients with T2D for all three metrics: AIRarg, AIRargMAX, and acute:MAX (**Figure 2**). As expected, AIRarg and AIRargMAX were both reduced, suggesting loss of functional and not fully functional BCM. The ratio acute:MAX was higher in T2D compared to HOV, suggesting that despite loss of both functional and not fully functional beta-cells, a higher proportion of beta-cells that were lost were those that require maximal stimulation and could possibly be categorized as not fully functional, stressed, or dormant (**Figure 2**).

In the whole pancreas, we found that a model with SUVR-1, as the only imaging metric, in combination with clinical biomarkers, was predictive of acute beta-cell function (AIRarg). SUVR-1, centroid, and principal axis length, together with clinical biomarkers, were predictive of maximum beta-cell function (AIRargMAX) in the whole pancreas. This suggests that, at least for T2D, the addition of MRI-based morphology metrics with SUVR-1 improves the prediction of structural and functional changes associated with loss of both functional and not fully functional beta-cells for the whole pancreas, compared to PET-only metrics (SUVR-1).

Previous histological findings demonstrated that T2D pancreata have greater rates of intralobular fibrosis and acinar to ductal metaplasia than non-diabetic pancreata (35). Therefore, unlike T1D, where drastic acinar cell volume loss occurs, acinar cells in T2D appear to remodel the pancreas through acinar to ductal metaplasia and increasing fibrosis, in agreement with less severe pancreas volume loss. The inclusion of all three centroid directions and principal axis length 3 suggests that acinar remodeling and fibrosis across the whole pancreas shift the pancreas center of mass but also shrink the pancreas to some extent along a short axis in T2D compared to HOV. Previous MRI metrics in T1D have noted that acinar atrophy typically occurs along the short axes, but the long axes remain mostly fixed due to the main duct running the length of the pancreas (30). Similarly, in our results, reduced principal axis length 3 was the only axis in the whole pancreas that was predictive of reduced AIRargMAX.

In our study, surface area was not predictive of AIRarg or AIRargMAX in our regression models and did not demonstrate group differences between HOV and T2D in the whole pancreas; however, the surface area of the pancreas body subregion was significantly lower in T2D (**Figure 7F**). Baseline pancreas volume and pancreas fractal dimension (similar to surface area) were significantly lower in T2D compared to non-diabetic controls (2, 4), and at 2-year follow-up, those with T2D remission had decreased pancreas fractal dimension and higher pancreas volume (4). A separate histological evaluation of the pancreas in T2D revealed that a majority of endocrine cell loss occurred in the head and tail with no significant changes in the body (36). In our dataset, MRI morphology metrics—principal axis length 3, EquivDiameter, bounding box 1, convex volume, and surface area—demonstrated group differences between HOV and T2D, but only in the pancreas body (**Figure 7**), suggesting that while limited endocrine loss may be occurring in the pancreas body (36), significant exocrine remodeling in the body may lead to changes visualized by such MRI morphology metrics. MRI of the pancreas has also been used in T2D to assess anterior-to-posterior diameter on axial slices, similar to our principal axis length 2 or 3 metrics. This method revealed significantly lower anterior-to-posterior pancreas diameters only for body and tail in short-term T2D, while long-term T2D had lower diameters in all regions (head, body, and tail) (32). Together, these suggest that exocrine changes in the pancreas may occur earlier and more severely in the body of the pancreas, although this remains to be studied longitudinally, both at the onset of disease and during treatment.

We performed exploratory correlations between single imaging metrics and either AIRarg or AIRargMAX. Pancreas head SUVR-1 and pancreas body principal axis length 3 were both significantly correlated to AIRarg and AIRargMAX (**Figure 8**). Typically, the highest proportion of beta-cells are lost from the head in T2D (36), and this was reflected in our previous report where the pancreas head SUVR-1 showed the largest differences between T2D and HOV (−17%) (3); however, principal axis length 3 in the pancreas body reflecting exocrine cell remodeling and loss in the pancreas body may also be predictive of endocrine cell loss (**Figure 8**).

VMAT2 and proinsulin have been shown to be co-expressed, and an increased amount of VMAT2/proinsulin expression was indicative of larger but dormant beta-cells (37), suggesting that VMAT2 may more accurately reflect an insulin vesicle functional capacity reservoir in non-functional and functional beta-cells and possibly hybrid alpha–beta-like cells (38). This may explain the ability of [^18^F]FP-(+)-DTBZ to capture functional and not fully functional beta-cell mass in this cohort.

Several studies have already shown the utility of pancreas MRI-based morphology metrics longitudinally in T1D and with the ability to predict outcomes (30, 31). Our current study was performed retrospectively in a cross-sectional cohort of HOV, prediabetes, and T2D, and it remains to be seen whether similar patterns and utility occur prospectively in both T2D and T1D when combining PET and MRI metrics. Similar to T1D, with MRI only to date, the combination of PET and MRI metrics could be used to assess BCM at diagnosis and to monitor therapeutic efficacy, where changes in the structure and function of the pancreas are more subtle and may require multi-modality imaging metrics. Alternatively, the combination of PET/MRI and longitudinal studies could provide PET-based validation of MRI-only metrics as surrogate markers of BCM, removing the need for PET studies, and reducing radiation exposure.

To our knowledge, this study is the first to combine PET imaging of BCM and MRI morphology metrics with a robust machine learning-based variable selection method to extract useful PET- and MRI-based metrics for predicting functional and not fully functional BCM. However, there are several limitations. Given the retrospective nature of the study, it is not possible to determine the temporal sequence of PET and MRI morphology metrics during progression to T2D. Thus, prospective longitudinal studies are necessary. This retrospective study had a relatively small sample size, although typical for PET imaging cohorts. The findings here need to be validated in larger, more diverse etiologies of T2D progression and treatment. Future investigations could also incorporate additional MR imaging to study MR relaxometry, quantitative fat fraction maps, diffusion-weighted imaging, perfusion imaging, and MR elastography, for example (28, 29), which would allow for further understanding of how changes in pancreas tissue composition drive the morphological changes we observed and how they relate to BCM assessed with PET imaging.

## Conclusion

Applying a robust machine learning-based variable selection method with a multi-modal imaging paradigm, as well as integrating PET with morphological metrics from MRI, provides a detailed assessment of functional and not fully functional BCM alterations in T2D. This approach explored a novel combination of biomarkers for staging of T2D and, in the future, possibly the evaluation of therapeutic interventions.

## Data Availability

The raw data supporting the conclusions of this article will be made available by the authors, without undue reservation.
