# Supplementary material for: Morphological and functional alterations in type 2 diabetes pancreata assessed with MRI-based metrics and [18F]FP-(+)-DTBZ PET
Source: Front Endocrinol (Lausanne). 2025 Dec 18;16:1724340. doi: 10.3389/fendo.2025.1724340 (PMC12756075; doi:10.3389/fendo.2025.1724340)
Supplement: Supplementary file 1 [file DataSheet1.pdf]

Supplementary Data

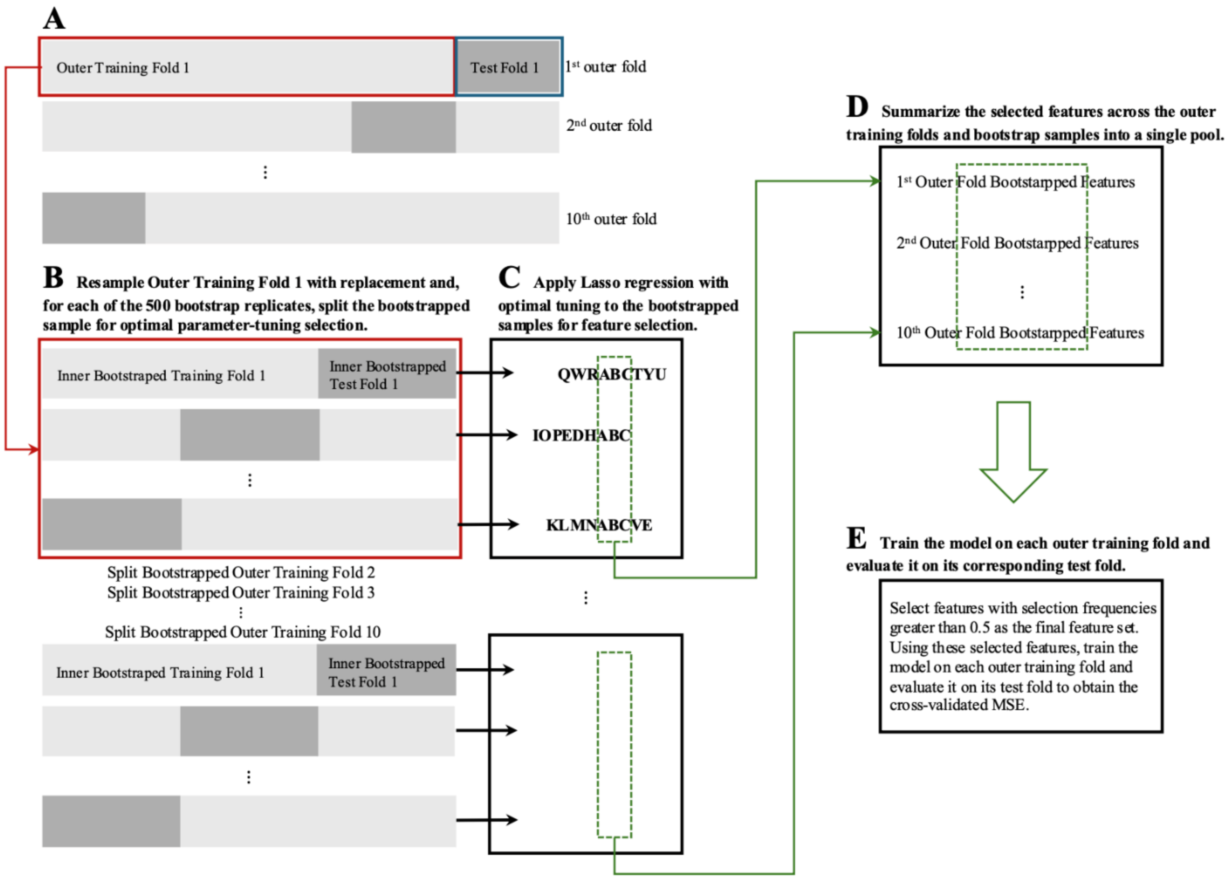

Supplementary Figure 1 Flowchart of implementation a fully nested cross-validation procedure in which both feature selection and model fitting are performed within each outer training fold. Specifically, for each outer fold, we generate 500 bootstrap resamples of the training data, carry out feature selection in each resample, and define "stable" predictors as those with overall selection frequency > 0.50 across all outer training folds and bootstrap iterations. Using these stable features, we then refit a linear regression model within each outer training fold and evaluate prediction error on the corresponding held-out test fold to obtain an unbiased estimate of the cross-validated MSE.

Supplementary Figure 2

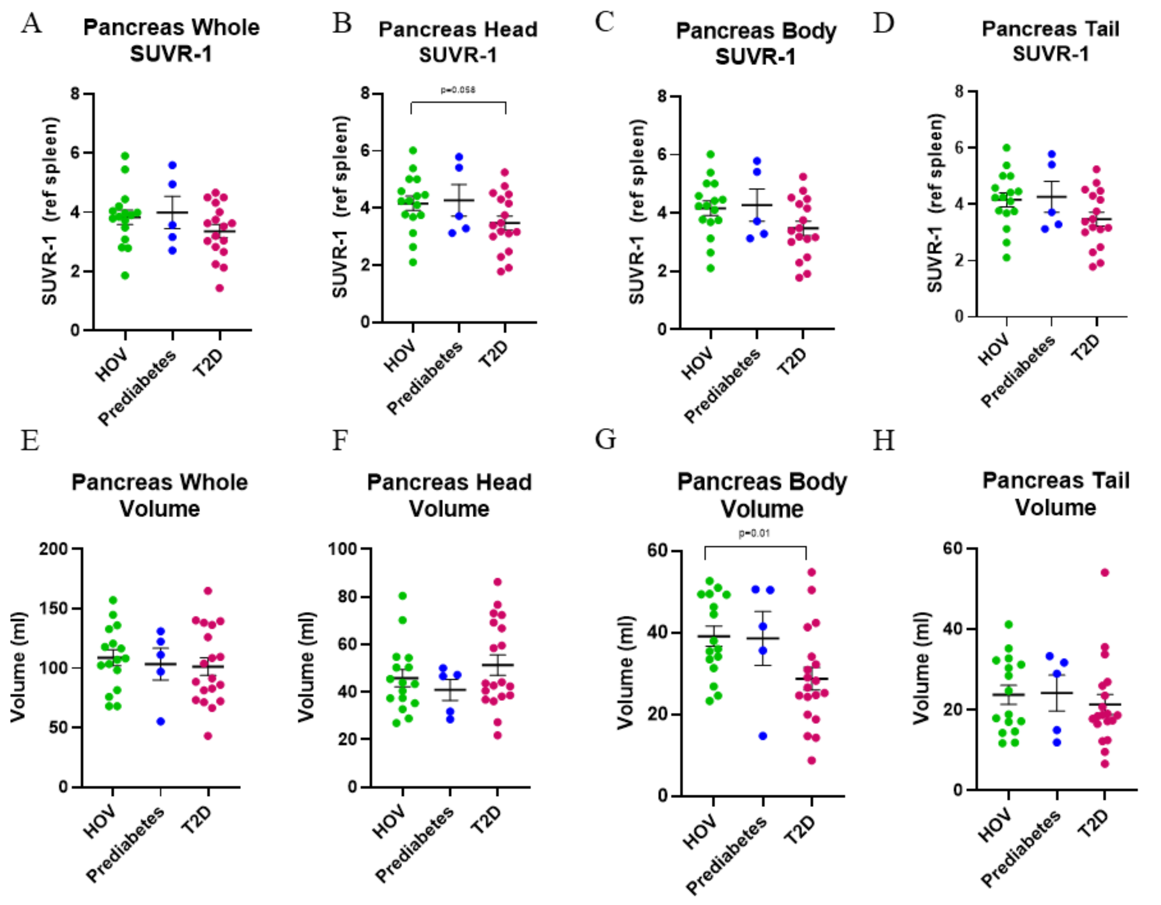

Group comparisons between healthy obese volunteers and individuals with T2D of the PET outcome metric SUVR-1 in pancreas **A)** whole **B)** head **C)** body and **D)** tail. Pancreas MRI volume metrics for pancreas **E)** whole **F)** head **G)** body and **H)** tail. All data presented as mean ± SEM.

Supplemental Table 1. Fitted full and reduced models for predicting primary functional beta cell mass outcomes (AIRarg, AIRargMAX and acute:MAX) with the primary PET outcome measure (SUVR-1), MRI morphology metrics and clinical covariates. Values in parentheses represent coefficient estimates; values in brackets denote 95% confidence intervals for the linear regression models; and “freq = ” indicates the selection frequencies of variables retained in each reduced model.

PET outcome measure	Pancreas ROI	Linear Models to Predict Acute Insulin Response to Arginine (AIRarg)	CV MSE
SUVR-1	Whole	AIRarg ~ **SUVR-1 (-0.43 [-1.49, 0.63])** + Volume (0.68 [-6.47, 7.83]) + Centroid1 (0.45 [-0.46, 1.36]) + Centroid2 (-0.11 [-1.10, 0.88]) + Centroid3 (0.70 [-0.24, 1.63]) + BoundingBox1 (-1.79 [-6.04, 2.46]) + BoundingBox2 (-3.14 [-8.48, 2.20]) + BoundingBox3 (-2.52 [-6.41, 1.37]) + BoundingBoxVolume (4.97 [-2.18, 12.12]) + EquivDiameter (6.19 [-4.19, 16.57]) + Extent (-0.54 [-3.91, 2.84]) + PrincipalAxisLength1 (0.50 [-2.27, 3.27]) + PrincipalAxisLength2 (-0.31 [-2.25, 1.63]) + PrincipalAxisLength3 (-0.07 [-1.59, 1.45]) + ConvexVolume (-5.76 [-14.82, 3.29]) + Solidity (-2.37 [-7.44, 2.69]) + SurfaceArea (-0.27 [-3.47, 2.94]) + **age (1.18 [-0.07, 2.44])** + gender (-0.67 [-2.95, 1.61]) + weight (1.01 [-0.56, 2.58]) + BMI (-1.37 [-3.26, 0.51]) + $HBA1_C$ (0.26 [-1.04, 1.57]) + **years.of.diabetes (-0.86 [-1.98, 0.26])** + diagnosisT2D (0.82 [-2.21, 3.85]) + diagnosispreDM (0.51 [-1.51, 2.54])	5.359
		AIRarg ~ SUVR-1 (0.21 [-0.16, 0.57], freq=0.61) + age (0.44 [0.08, 0.79], freq=0.78) + years.of.diabetes (-0.64 [-1.04, -0.25], freq=0.77)	1.264
	Head	AIRarg ~ **SUVR-1 (0.13 [-0.82, 1.07])** + Volume (-4.45 [-17.02, 8.13]) + **Centroid1 (-0.47 [-1.35, 0.42])** + Centroid2 (-0.03 [-1.37, 1.30]) + Centroid3 (-0.22 [-1.58, 1.15]) + BoundingBox1 (-1.8 [-7.42, 3.82]) + BoundingBox2 (-1.49 [-8.57, 5.58]) + BoundingBox3 (-1.58 [-6.81, 3.65]) + BoundingBoxVolume (1.63 [-12.7, 15.96]) + EquivDiameter (-0.54 [-13.09, 12.02]) + Extent (-0.48 [-6.01, 5.06]) + PrincipalAxisLength1 (-0.15 [-2.7, 2.41]) + PrincipalAxisLength2 (-0.72 [-4.1, 2.65]) + PrincipalAxisLength3 (-0.94 [-4.47, 2.59]) + ConvexVolume (8.8 [-5.68, 23.28]) + Solidity (1.38 [-3.7, 6.46]) + SurfaceArea (-0.99 [-3.84, 1.86]) + **age (0.50 [-1.07, 2.07])** + gender (-0.67 [-2.44, 1.09]) + weight (0.03 [-1.89, 1.95]) + BMI (0.21 [-1.75, 2.16]) + $HBA1_C$ (-0.46 [-1.89, 0.97]) + **years.of.diabetes (-0.65 [-1.82, 0.51])** + diagnosisT2D (1.07 [-1.92, 4.06]) + diagnosispreDM (1.04 [-1.96, 4.05])	5.666
		AIRarg ~ SUVR-1 (0.22 [-0.16, 0.6], freq=0.64) + Centroid1 (-0.15 [-0.51, 0.2], freq=0.55) + age (0.48 [0.12, 0.85], freq=0.78) + years.of.diabetes (-0.61 [-1.02, -0.21], freq=0.77)	1.231
	Body	AIRarg ~ **SUVR-1 (0.22 [-0.58, 1.03])** + Volume (-0.60 [-8.44, 7.25]) + **Centroid1 (0.53 [-0.75, 1.81])** + **Centroid2 (-0.26 [-1.09, 0.56])** + Centroid3 (-0.02 [-0.73, 0.7]) + BoundingBox1 (1.35 [-8.3, 11.01]) + BoundingBox2 (0.58 [-8.68, 9.85]) + BoundingBox3 (-0.04 [-8.39, 8.31]) + BoundingBoxVolume (1.66 [-6.49, 9.82]) + EquivDiameter (1.77 [-11.81, 15.34]) + Extent (1.02 [-5.09, 7.13]) + PrincipalAxisLength1 (-0.55 [-5.13, 4.03]) + PrincipalAxisLength2 (0.47 [-1.37, 2.31]) + **PrincipalAxisLength3 (0.78 [-2.03, 3.59])** + ConvexVolume (-4.09 [-14.41, 6.23]) + **Solidity (-1.21 [-4.48, 2.05])** + SurfaceArea (-0.79 [-2.27, 0.70]) + **age (0.15 [-0.86, 1.17])** + gender (-0.48 [-2.79, 1.82]) + weight (0.02 [-1.24, 1.28]) + **BMI (0.29 [-0.84,**	6.623

		**1.43])** + HBA1$_C$ (-0.43 [-1.91, 1.05]) + **years.of.diabetes (-0.49 [-2.00, 1.02])** + diagnosisT2D (0.90 [-1.80, 3.59]) + diagnosispreDM (1.06 [-1.50, 3.62])	
		AIRarg ~ SUVR-1 (0.15 [-0.25, 0.55], freq=0.71) + Centroid1 (-0.10 [-0.49, 0.28], freq=0.50) + Centroid2 (0.02 [-0.37, 0.42], freq=0.56) + PrincipalAxisLength3 (0.34 [-0.09, 0.77], freq=0.69) + Solidity (-0.18 [-0.57, 0.21], freq=0.61) + age (0.47 [0.07, 0.87], freq=0.77) + BMI (0.2 [-0.17, 0.58], freq=0.52) + years.of.diabetes (-0.54 [-0.98, -0.11], freq=0.77)	1.395
	Tail	AIRarg ~ **SUVR-1 (0.17 [-0.68, 1.02])** + Volume (0.28 [-5.35, 5.91]) + Centroid1 (-0.01 [-0.84, 0.82]) + **Centroid2 (0.16 [-0.76, 1.08])** + **Centroid3 (0.02 [-0.84, 0.87])** + **BoundingBox1 (2.55 [-1.86, 6.95])** + BoundingBox2 (1.79 [-2.44, 6.03]) + BoundingBox3 (0.79 [-3.23, 4.81]) + BoundingBoxVolume (-0.16 [-7.17, 6.85]) + EquivDiameter (-1.59 [-9.05, 5.87]) + Extent (0.38 [-3.08, 3.84]) + PrincipalAxisLength1 (-1.26 [-4.23, 1.71]) + PrincipalAxisLength2 (-0.19 [-2.18, 1.81]) + **PrincipalAxisLength3 (0.72 [-1.99, 3.43])** + ConvexVolume (-0.22 [-9.58, 9.14]) + Solidity (0.70 [-2.55, 3.94]) + SurfaceArea (-1.10 [-2.28, 0.07]) + **age (0.55 [-0.26, 1.36])** + gender (-0.45 [-2.11, 1.21]) + weight (0.16 [-0.79, 1.11]) + **BMI (0.16 [-0.76, 1.08])** + **HBA1$_C$ (-0.05 [-1.13, 1.02])** + **years.of.diabetes (-0.47 [-1.40, 0.45])** + diagnosisT2D (0.47 [-1.48, 2.42]) + diagnosispreDM (0.8 [-0.82, 2.43])	4.370
		AIRarg ~ SUVR1 (0.20 [-0.22, 0.61], freq=0.59) + Centroid2 (-0.11 [-0.54, 0.32], freq=0.50) + Centroid3 (0.12 [-0.26, 0.50], freq=0.55) + BoundingBox1 (0.25 [-0.18, 0.67], freq=0.65) + PrincipalAxisLength3 (0.22 [-0.19, 0.63], freq=0.54) + age (0.56 [0.16, 0.96], freq=0.86) + BMI (0.21 [-0.18, 0.59], freq=0.60) + HBA1$_C$ (0.04 [-0.50, 0.59], freq=0.50) + years.of.diabetes (-0.61 [-1.23, 0.02], freq=0.80)	1.244
		**Linear Models to Predict maximum insulin response to arginine (AIRargMAX)**	
SUVR-1	Whole	AIRargMAX ~ **SUVR-1 (0.03 [-2.09, 2.15])** + Volume (3.43 [-10.87, 17.73]) + **Centroid1 (-0.03 [-1.86, 1.8])** + **Centroid2 (-1.99 [-3.97, -0.01])** + **Centroid3 (1.47 [-0.40, 3.34])** + BoundingBox1 (-5.5 [-14, 2.99]) + BoundingBox2 (-4.41 [-15.09, 6.26]) + BoundingBox3 (-3.78 [-11.56, 3.99]) + BoundingBoxVolume (1.28 [-13.02, 15.58]) + EquivDiameter (0.64 [-20.12, 21.39]) + Extent (-3.74 [-10.49, 3.00]) + PrincipalAxisLength1 (2.72 [-2.83, 8.26]) + PrincipalAxisLength2 (-0.26 [-4.14, 3.61]) + **PrincipalAxisLength3 (0.23 [-2.81, 3.27])** + ConvexVolume (0.76 [-17.36, 18.87]) + Solidity (0.66 [-9.46, 10.79]) + SurfaceArea (1.64 [-4.78, 8.05]) + **age (1.42 [-1.09, 3.93])** + gender (-2.83 [-7.39, 1.73]) + weight (0.98 [-2.17, 4.12]) + **BMI (-1.41 [-5.19, 2.36])** + **HBA1$_C$ (-1.69 [-4.3, 0.92])** + **years.of.diabetes (-1.11 [-3.35, 1.12**]) + diagnosisT2D (0.1 [-5.95, 6.16]) + diagnosispreDM (-2.10 [-6.16, 1.96])	22.872
		AIRargMAX ~ SUVR-1 (0.67 [-0.22, 1.56], freq=0.79) + Centroid1 (-0.23 [-1.19, 0.74], freq=0.54) + Centroid2 (-1.11 [-2.03, -0.18], freq=0.81) + Centroid3 (1.29 [0.38, 2.20], freq=0.85) + PrincipalAxisLength3 (0.58 [-0.3, 1.46], freq=0.58) + age (0.55 [-0.33, 1.43], freq=0.59) + BMI (-0.66 [-1.56, 0.23], freq=0.53) + HBA1$_C$ (-1.81 [-3.03, -0.59], freq=0.97) + years.of.diabetes (-1.05 [-2.38, 0.29], freq=0.80)	8.582
	Head	AIRargMAX ~ **SUVR-1 (-0.02 [-1.8, 1.76])** + Volume (-2.86 [-26.65, 20.94]) + **Centroid1 (-0.60 [-2.27, 1.08])** + **Centroid2 (-0.88 [-3.4, 1.65])** + **Centroid3 (0.75 [-1.83, 3.34])** + BoundingBox1 (-8.93 [-19.56, 1.71]) +	20.432

		BoundingBox2 (-9.7 [-23.09, 3.69]) + BoundingBox3 (-8.15 [-18.05, 1.74]) + BoundingBoxVolume (9.81 [-17.3, 36.93]) + EquivDiameter (13.83 [-9.93, 37.6]) + Extent (-3.41 [-13.88, 7.06]) + **PrincipalAxisLength1 (-0.99 [-5.82, 3.85])** + **PrincipalAxisLength2 (-3.65 [-10.04, 2.74])** + **PrincipalAxisLength3 (-3.23 [-9.91, 3.46])** + ConvexVolume (6.38 [-21.03, 33.79]) + Solidity (-2.12 [-11.74, 7.50]) + SurfaceArea (-1.83 [-7.22, 3.57]) + **age (1.26 [-1.71, 4.24])** + gender (-0.58 [-3.91, 2.76]) + weight (0.48 [-3.16, 4.12]) + BMI (-0.83 [-4.53, 2.87]) + **HBA1c (-1.04 [-3.74, 1.67])** + **years.of.diabetes (-1.29 [-3.5, 0.92])** + diagnosisT2D (-1.61 [-7.27, 4.04]) + diagnosispreDM (0.75 [-4.94, 6.44])	
		AIRargMAX ~ SUVR-1 (0.39 [-0.57, 1.35], freq=0.70) + Centroid1 (-0.29 [-1.32, 0.75], freq=0.58) + Centroid2 (-1 [-2.1, 0.11], freq=0.83) + Centroid3 (0.66 [-0.34, 1.66], freq=0.71) + PrincipalAxisLength1 (0.39 [-0.60, 1.39], freq=0.51) + PrincipalAxisLength2 (-0.85 [-1.96, 0.26], freq=0.85) + PrincipalAxisLength3 (0.53 [-0.44, 1.50], freq=0.62) + age (0.53 [-0.43, 1.49], freq=0.62) + HBA1c (-1.09 [-2.48, 0.30], freq=0.87) + years.of.diabetes (-1.53 [-2.88, -0.18], freq=0.87)	8.669
	Body	AIRargMAX ~ **SUVR-1 (-0.17 [-1.65, 1.32])** + Volume (7.12 [-7.32, 21.56]) + **Centroid1 (-1.23 [-3.59, 1.12])** + **Centroid2 (-0.84 [-2.36, 0.67])** + **Centroid3 (1.26 [-0.06, 2.57])** + BoundingBox1 (13.1 [-4.67, 30.87]) + BoundingBox2 (10.89 [-6.15, 27.94]) + BoundingBox3 (7.63 [-7.74, 22.99]) + BoundingBoxVolume (-6.41 [-21.42, 8.6]) + EquivDiameter (-16.06 [-41.04, 8.93]) + Extent (5.74 [-5.5, 16.99]) + **PrincipalAxisLength1 (-5.24 [-13.66, 3.18])** + **PrincipalAxisLength2 (0.25 [-3.14, 3.64])** + **PrincipalAxisLength3 (-0.21 [-5.37, 4.96])** + ConvexVolume (1.41 [-17.58, 20.41]) + Solidity (0.6 [-5.41, 6.6]) + SurfaceArea (-1.5 [-4.23, 1.23]) + **age (0.79 [-1.08, 2.66])** + gender (-0.98 [-5.22, 3.26]) + **weight (-1.06 [-3.37, 1.26])** + BMI (0.86 [-1.23, 2.95]) + **HBA1c (-1.32 [-4.04, 1.41])** + **years.of.diabetes (0.18 [-2.60, 2.96])** + diagnosisT2D (-4.89 [-9.84, 0.07]) + diagnosispreDM (-2.38 [-7.08, 2.33])	21.252
		AIRargMAX ~ SUVR-1 (0.15 [-0.68, 0.98], freq=0.72) + Centroid1 (-0.43 [-1.34, 0.48], freq=0.61) + Centroid2 (-0.92 [-1.78, -0.05], freq=0.85) + Centroid3 (1.45 [0.50, 2.39], freq=0.84) + PrincipalAxisLength1 (-0.25 [-1.25, 0.75], freq=0.52) + PrincipalAxisLength2 (0.72 [-0.26, 1.71], freq=0.56) + PrincipalAxisLength3 (1.16 [0.10, 2.22], freq=0.80) + age (0.68 [-0.26, 1.62], freq=0.69) + weight (-0.45 [-1.33, 0.43], freq=0.51) + HBA1c (-2.11 [-3.32, -0.91], freq=0.95) + years.of.diabetes (-0.58 [-1.89, 0.72], freq=0.72)	6.569
	Tail	AIRargMAX ~ **SUVR-1 (0.88 [-1.41, 3.17])** + Volume (2.59 [-12.58, 17.77]) + **Centroid1 (0.04 [-2.19, 2.27])** + **Centroid2 (-0.92 [-3.39, 1.55])** + **Centroid3 (1.61 [-0.68, 3.91])** + BoundingBox1 (2.42 [-9.44, 14.29]) + BoundingBox2 (1.24 [-10.17, 12.66]) + BoundingBox3 (2.99 [-7.84, 13.82]) + BoundingBoxVolume (1.84 [-17.04, 20.72]) + EquivDiameter (-5.36 [-25.45, 14.72]) + Extent (2 [-7.31, 11.31]) + PrincipalAxisLength1 (-0.11 [-8.11, 7.88]) + PrincipalAxisLength2 (1.16 [-4.21, 6.52]) + **PrincipalAxisLength3 (0.9 [-6.4, 8.21])** + ConvexVolume (-2.9 [-28.1, 22.3]) + Solidity (0.1 [-8.64, 8.85]) + SurfaceArea (-1.38 [-4.55, 1.78]) + **age (0.87 [-1.3, 3.05])** + gender (-0.28 [-4.74, 4.18]) + weight (0.16 [-2.39, 2.72]) + BMI (0.12 [-2.37, 2.6]) + **HBA1c (-1.16 [-4.05, 1.74])** + **years.of.diabetes (-0.62 [-3.12, 1.87])** + diagnosisT2D (-3.43 [-8.68, 1.82]) + diagnosispreDM (-1.79 [-6.18, 2.59])	30.362

		AIRargMAX ~ SUVR-1 (0.54 [-0.44, 1.52], freq=0.68) + Centroid1 (-0.44 [-1.56, 0.69], freq=0.68) + Centroid2 (-0.71 [-1.78, 0.36], freq=0.63) + Centroid3 (0.84 [-0.18, 1.87], freq=0.75) + PrincipalAxisLength3 (0.43 [-0.5, 1.37], freq=0.5) + age (0.53 [-0.42, 1.47], freq=0.56) + HBA1$_C$ (-1.93 [-3.17, -0.7], freq=0.95) + years.of.diabetes (-1.2 [-2.59, 0.18], freq=0.81)	7.713
		**Linear Models to Predict ratio of acute to maximum insulin response to arginine (acute:MAX)**	
SUVR-1	Whole	acute:MAX ~ **SUVR-1 (-0.02 [-0.13, 0.08])** + Volume (0.01 [-0.69, 0.7]) + **Centroid1 (0.06 [-0.03, 0.15])** + **Centroid2 (0.05 [-0.05, 0.15])** + **Centroid3 (0.05 [-0.05, 0.14])** + **BoundingBox1 (0.22 [-0.19, 0.64])** + BoundingBox2 (0.1 [-0.42, 0.62]) + **BoundingBox3 (0.11 [-0.27, 0.49])** + BoundingBoxVolume (0.31 [-0.39, 1.01]) + EquivDiameter (0.32 [-0.69, 1.34]) + Extent (0.33 [0, 0.66]) + **PrincipalAxisLength1 (0.03 [-0.24, 0.30])** + **PrincipalAxisLength2 (0.08 [-0.11, 0.27])** + **PrincipalAxisLength3 (0.02 [-0.13, 0.17])** + ConvexVolume (-0.71 [-1.59, 0.18]) + Solidity (-0.26 [-0.75, 0.24]) + SurfaceArea (-0.19 [-0.5, 0.13]) + **age (0.07 [-0.05, 0.20])** + gender (0.03 [-0.2, 0.25]) + weight (0.04 [-0.11, 0.19]) + **BMI (-0.07 [-0.26, 0.11])** + **HBA1$_C$ (0.24 [0.11, 0.36])** + **years.of.diabetes (-0.11 [-0.22, 0.00])** + diagnosisT2D (-0.03 [-0.32, 0.27]) + diagnosispreDM (0.07 [-0.13, 0.26])	0.060
		acute:MAX ~ SUVR-1 (0.02 [-0.04, 0.07], freq=0.62) + Centroid1 (0.03 [-0.03, 0.09], freq=0.73) + Centroid2 (0.01 [-0.06, 0.07], freq=0.55) + Centroid3 (-0.01 [-0.06, 0.05], freq=0.57) + BoundingBox1 (0.05 [-0.07, 0.16], freq=0.57) + BoundingBox3 (0.06 [0, 0.12], freq=0.66) + PrincipalAxisLength1 (0.02 [-0.09, 0.13], freq=0.58) + PrincipalAxisLength2 (-0.03 [-0.08, 0.02], freq=0.51) + PrincipalAxisLength3 (-0.09 [-0.16, -0.03], freq=0.69) + age (0.05 [0, 0.11], freq=0.71) + BMI (0.01 [-0.04, 0.06], freq=0.51) + HBA1$_C$ (0.23 [0.15, 0.3], freq=0.98) + years.of.diabetes (-0.12 [-0.2, -0.04], freq=0.63)	0.026
	Head	acute:MAX ~ **SUVR-1 (0 [-0.13, 0.14])** + Volume (-0.49 [-2.31, 1.32]) + Centroid1 (-0.05 [-0.18, 0.08]) + Centroid2 (-0.04 [-0.24, 0.15]) + Centroid3 (0.05 [-0.14, 0.25]) + BoundingBox1 (0.45 [-0.36, 1.26]) + BoundingBox2 (0.49 [-0.53, 1.51]) + BoundingBox3 (0.36 [-0.39, 1.12]) + BoundingBoxVolume (-1.31 [-3.38, 0.75]) + EquivDiameter (-0.23 [-2.04, 1.58]) + Extent (-0.28 [-1.07, 0.52]) + **PrincipalAxisLength1 (0.05 [-0.32, 0.42])** + **PrincipalAxisLength2 (0.19 [-0.3, 0.67])** + **PrincipalAxisLength3 (-0.02 [-0.53, 0.49])** + ConvexVolume (1.06 [-1.03, 3.15]) + Solidity (0.39 [-0.34, 1.13]) + SurfaceArea (-0.18 [-0.59, 0.23]) + **age (0.14 [-0.09, 0.37])** + gender (0 [-0.26, 0.25]) + weight (0.05 [-0.23, 0.32]) + BMI (-0.01 [-0.29, 0.27]) + **HBA1$_C$ (0.17 [-0.03, 0.38])** + **years.of.diabetes (-0.12 [-0.29, 0.04])** + diagnosisT2D (0.06 [-0.37, 0.5]) + diagnosispreDM (-0.16 [-0.59, 0.28])	0.113
		acute:MAX ~ SUVR-1 (0.04 [-0.01, 0.09], freq=0.59) + PrincipalAxisLength1 (-0.02 [-0.07, 0.03], freq=0.54) + PrincipalAxisLength2 (0.03 [-0.02, 0.09], freq=0.62) + PrincipalAxisLength3 (-0.04 [-0.09, 0.01], freq=0.61) + age (0.05 [0, 0.10], freq=0.63) + HBA1$_C$ (0.21 [0.13, 0.28], freq=0.97) + years.of.diabetes (-0.10 [-0.17, -0.02], freq=0.56)	0.026
	Body	acute:MAX ~ **SUVR-1 (0.03 [-0.03, 0.1])** + Volume (-0.34 [-0.99, 0.31]) + **Centroid1 (0.08 [-0.02, 0.19])** + **Centroid2 (-0.04 [-0.11, 0.03]) + Centroid3 (0.03 [-0.02, 0.09])** + **BoundingBox1 (-0.48 [-1.28, 0.32])** + **BoundingBox2 (-0.47 [-1.24, 0.29])** + **BoundingBox3 (-0.32 [-1.01, 0.37])** + BoundingBoxVolume (0.31 [-0.36, 0.99]) + EquivDiameter (0.63 [-0.49,	0.031

		1.75]) + **Extent (-0.27 [-0.77, 0.24])** + PrincipalAxisLength1 (0.37 [-0.01, 0.75]) + **PrincipalAxisLength2 (0.04 [-0.11, 0.19])** + PrincipalAxisLength3 (0.2 [-0.03, 0.43]) + ConvexVolume (-0.16 [-1.02, 0.69]) + **Solidity (-0.01 [-0.28, 0.26])** + SurfaceArea (-0.09 [-0.21, 0.03]) + **age (0.07 [-0.02, 0.15])** + gender (0.13 [-0.06, 0.33]) + weight (0.05 [-0.06, 0.15]) + **BMI (-0.03 [-0.13, 0.06])** + **HBA1$_C$ (0.23 [0.11, 0.35])** + **years.of.diabetes (-0.2 [-0.32, -0.07])** + diagnosisT2D (0.22 [0, 0.45]) + diagnosispreDM (0.13 [-0.08, 0.34])	
		acute:MAX ~ SUVR-1 (0.04 [-0.02, 0.10], freq=0.72) + Centroid1 (0.04 [-0.03, 0.11], freq=0.7) + Centroid2 (-0.02 [-0.07, 0.04], freq=0.64) + Centroid3 (0.02 [-0.04, 0.08], freq=0.64) + BoundingBox1 (0.05 [-0.03, 0.13], freq=0.65) + BoundingBox2 (-0.02 [-0.09, 0.05], freq=0.53) + BoundingBox3 (0.01 [-0.06, 0.09], freq=0.59) + Extent (-0.05 [-0.16, 0.06], freq=0.61) + PrincipalAxisLength2 (-0.07 [-0.15, 0], freq=0.72) + Solidity (0.02 [-0.07, 0.1], freq=0.56) + age (0.07 [0.00, 0.14], freq=0.73) + weight (0.06 [-0.04, 0.15], freq=0.56) + BMI (-0.04 [-0.13, 0.04], freq=0.55) + HBA1$_C$ (0.25 [0.18, 0.33], freq=0.99) + years.of.diabetes (-0.13 [-0.21, -0.04], freq=0.68)	0.024
	Tail	acute:MAX ~ **SUVR-1 (0.01 [-0.11, 0.13])** + Volume (0.09 [-0.69, 0.87]) + **Centroid1 (0.04 [-0.08, 0.15])** + **Centroid2 (0.07 [-0.06, 0.2])** + **Centroid3 (0 [-0.12, 0.12])** + BoundingBox1 (0.44 [-0.17, 1.05]) + BoundingBox2 (0.43 [-0.15, 1.02]) + BoundingBox3 (0.27 [-0.28, 0.83]) + BoundingBoxVolume (-0.61 [-1.58, 0.36]) + EquivDiameter (-0.63 [-1.66, 0.4]) + Extent (0.04 [-0.43, 0.52]) + PrincipalAxisLength1 (-0.05 [-0.46, 0.36]) + **PrincipalAxisLength2 (-0.01 [-0.29, 0.26])** + PrincipalAxisLength3 (0.17 [-0.2, 0.55]) + ConvexVolume (0.29 [-1.00, 1.59]) + Solidity (0.21 [-0.24, 0.65]) + SurfaceArea (-0.04 [-0.2, 0.12]) + **age (0.08 [-0.03, 0.19])** + gender (-0.02 [-0.25, 0.21]) + weight (0.07 [-0.06, 0.20]) + BMI (-0.06 [-0.19, 0.07]) + **HBA1$_C$ (0.22 [0.07, 0.37])** + **years.of.diabetes (-0.12 [-0.25, 0.00])** + diagnosisT2D (0.13 [-0.14, 0.40]) + diagnosispreDM (0.15 [-0.08, 0.37])	0.069
		acute:MAX ~ SUVR-1 (0.03 [-0.03, 0.09], freq=0.51) + Centroid1 (0.06 [0, 0.12], freq=0.72) + Centroid2 (0 [-0.06, 0.07], freq=0.56) + Centroid3 (0.02 [-0.03, 0.08], freq=0.52) + PrincipalAxisLength2 (-0.04 [-0.10, 0.02], freq=0.61) + age (0.05 [0, 0.11], freq=0.68) + HBA1$_C$ (0.21 [0.14, 0.28], freq=0.97) + years.of.diabetes (-0.10 [-0.18, -0.02], freq=0.59)	0.025

Supplementary Table 2 Fitted full and reduced models for predicting the primary functional beta-cell mass outcome (AIRarg) using the primary PET outcome measures (SUVR-1 × Volume, BPnd, and BPnd × Volume), MRI morphology metrics, and clinical covariates. Values in parentheses represent coefficient estimates; values in brackets denote 95% confidence intervals; and "freq = " indicates the selection frequencies of variables retained in each reduced model.

PET outcome measure	Pancreas ROI	Linear Models to Predict Acute Insulin Response to Arginine (AIRarg) Linear Models	CV MSE
SUVR-1 x Volume	Whole	AIRarg ~ SUVR-1 x Volume (-0.74 [-2.11, 0.64]) + Volume (2.05 [-5.81, 9.92]) + **Centroid1 (0.46 [-0.28, 1.2])** + Centroid2 (-0.1 [-1.03, 0.82]) + **Centroid3 (0.62 [-0.16, 1.4])** + BoundingBox1 (-1.95 [-5.79, 1.89]) +	4.470

		BoundingBox2 (-3.2 [-8.05, 1.65]) + BoundingBox3 (-2.6 [-6.17, 0.98]) + BoundingBoxVolume (4.89 [-1.62, 11.39]) + EquivDiameter (5.1 [-4, 14.19]) + Extent (-0.64 [-3.78, 2.5]) + PrincipalAxisLength1 (0.57 [-1.76, 2.91]) + PrincipalAxisLength2 (-0.31 [-2.02, 1.41]) + PrincipalAxisLength3 (-0.07 [-1.42, 1.27]) + ConvexVolume (-5.66 [-13.77, 2.44]) + Solidity (-2.35 [-6.95, 2.25]) + SurfaceArea (-0.07 [-2.96, 2.81]) + **age (1.12 [0.06, 2.19])** + gender (-0.88 [-2.8, 1.03]) + weight (1.02 [-0.39, 2.43]) + BMI (-1.37 [-3.01, 0.27]) + HBA1$_{C}$ (0.22 [-0.98, 1.43]) + **years.of.diabetes (-0.83 [-1.84, 0.18])** + diagnosisT2D (0.84 [-1.94, 3.62]) + diagnosispreDM (0.51 [-1.39, 2.40])	
		AIRarg ~ Centroid1 (0.13 [-0.23, 0.48], freq=0.52) + Centroid3 (0.15 [-0.2, 0.49], freq=0.52) + age (0.45 [0.09, 0.8], freq=0.84) + years.of.diabetes (-0.7 [-1.08, -0.33], freq=0.84)	2.092
	Head	AIRarg ~ **SUVR-1 x Volume (0.04 [-1.52, 1.6])** + Volume (-5.29 [-16.38, 5.81]) + **Centroid1 (-0.46 [-1.3, 0.38])** + Centroid2 (-0.11 [-1.36, 1.14]) + Centroid3 (-0.11 [-1.5, 1.28]) + BoundingBox1 (-1.76 [-6.98, 3.45]) + BoundingBox2 (-1.5 [-8.1, 5.09]) + BoundingBox3 (-1.54 [-6.46, 3.38]) + BoundingBoxVolume (0.88 [-13.04, 14.80]) + EquivDiameter (0.17 [-11.93, 12.27]) + Extent (-0.88 [-5.97, 4.20]) + PrincipalAxisLength1 (-0.08 [-2.19, 2.02]) + PrincipalAxisLength2 (-0.64 [-3.58, 2.3]) + PrincipalAxisLength3 (-0.99 [-4.16, 2.19]) + ConvexVolume (9.56 [-4.04, 23.17]) + Solidity (1.68 [-3.02, 6.38]) + SurfaceArea (-1.03 [-3.74, 1.69]) + **age (0.62 [-0.89, 2.14])** + gender (-0.63 [-2.27, 1.01]) + weight (0.16 [-1.70, 2.03]) + BMI (0.07 [-1.85, 1.98]) + HBA1$_{C}$ (-0.44 [-1.75, 0.86]) + **years.of.diabetes (-0.72 [-1.74, 0.29])** + diagnosisT2D (1.12 [-1.68, 3.92]) + diagnosis.preDM (0.94 [-1.98, 3.86])	4.868
		AIRarg ~ SUVR-1 x Volume (0.17 [-0.21, 0.55], freq=0.52) + Centroid1 (-0.12 [-0.47, 0.23], freq=0.56) + age (0.51 [0.14, 0.88], freq=0.82) + years.of.diabetes (-0.71 [-1.07, -0.34], freq=0.84)	2.133
	Body	AIRarg ~ SUVR-1 x Volume (0.04 [-1.24, 1.33]) + Volume (0.09 [-7.80, 7.99]) + **Centroid1 (0.57 [-0.52, 1.66])** + **Centroid2 (-0.22 [-0.99, 0.56])** + **Centroid3 (0.01 [-0.67, 0.69])** + BoundingBox1 (2.81 [-6.32, 11.93]) + BoundingBox2 (2.12 [-6.57, 10.82]) + BoundingBox3 (1.38 [-6.42, 9.19]) + BoundingBoxVolume (0.28 [-7.74, 8.29]) + EquivDiameter (-0.01 [-13.36, 13.34]) + Extent (1.86 [-3.77, 7.49]) + PrincipalAxisLength1 (-1.02 [-5.34, 3.29]) + PrincipalAxisLength2 (0.21 [-1.46, 1.87]) + **PrincipalAxisLength3 (0.42 [-2.14, 2.97])** + ConvexVolume (-3.67 [-13.52, 6.17]) + **Solidity (-1.22 [-4.13, 1.69])** + **SurfaceArea (-0.88 [-2.27, 0.51])** + **age (0.19 [-0.79, 1.16])** + gender (-0.27 [-2.44, 1.91]) + weight (0.06 [-1.01, 1.13]) + **BMI (0.21 [-0.75, 1.17])** + HBA1$_{C}$ (-0.32 [-1.8, 1.16]) + **years.of.diabetes (-0.58 [-2.04, 0.87])** + diagnosisT2D (0.63 [-2.09, 3.35]) + diagnosispreDM (1.28 [-1.02, 3.58])	4.999
		AIRarg ~ Centroid1 (0.21 [-0.19, 0.62], freq=0.54) + Centroid2 (-0.06 [-0.43, 0.31], freq=0.54) + Centroid3 (0.11 [-0.31, 0.53], freq=0.54) + PrincipalAxisLength3 (0.63 [0.21, 1.04], freq=0.74) + Solidity (-0.39 [-0.77, -0.01], freq=0.64) + SurfaceArea (-0.6 [-1.14, -0.07], freq=0.51) + age (0.34 [-0.04, 0.73], freq=0.81) + BMI (0.14 [-0.22, 0.5], freq=0.51) + years.of.diabetes (-0.68 [-1.09, -0.28], freq=0.85)	2.183
	Tail	AIRarg ~ SUVR-1 x Volume (-0.08 [-1.53, 1.37]) + Volume (1.31 [-5.34, 7.95]) + **Centroid1 (0.03 [-0.68, 0.74])** + Centroid2 (0.26 [-0.65, 1.17]) + **Centroid3 (-0.02 [-0.85, 0.81])** + **BoundingBox1 (1.92 [-2.35, 6.18])** +	4.964

		BoundingBox2 (1.25 [-2.87, 5.38]) + BoundingBox3 (0.36 [-3.6, 4.32]) + BoundingBoxVolume (0.78 [-6.09, 7.66]) + EquivDiameter (-1.18 [-8.27, 5.91]) + Extent (0.23 [-3.1, 3.56]) + PrincipalAxisLength1 (-0.92 [-3.77, 1.92]) + PrincipalAxisLength2 (-0.1 [-2.01, 1.82]) + **PrincipalAxisLength3 (0.79 [-1.82, 3.39])** + ConvexVolume (-1.6 [-10.83, 7.64]) + Solidity (0.41 [-2.64, 3.47]) + SurfaceArea (-1.22 [-2.34, -0.1]) + **age (0.59 [-0.18, 1.36])** + gender (-0.52 [-2.02, 0.99]) + weight (0.19 [-0.67, 1.05]) + **BMI (0.06 [-0.77, 0.89])** + $HBA1_C$ (-0.15 [-1.21, 0.90]) + **years.of.diabetes (-0.6 [-1.43, 0.23])** + diagnosisT2D (0.62 [-1.22, 2.47]) + diagnosispreDM (0.77 [-0.77, 2.3])	
		AIRarg ~ Centroid1 (0.03 [-0.38, 0.45], freq=0.55) + Centroid3 (0.13 [-0.28, 0.54], freq=0.53) + BoundingBox1 (0.19 [-0.22, 0.59], freq=0.64) + PrincipalAxisLength3 (0.29 [-0.11, 0.69], freq=0.56) + age (0.55 [0.19, 0.92], freq=0.89) + BMI (0.18 [-0.21, 0.57], freq=0.59) + years.of.diabetes (-0.67 [-1.03, -0.31], freq=0.87)	2.174
BP_{ND}	Whole	AIRarg ~ **BP_{ND} (-2.08 [-4.28, 0.11])** + Volume (4.11 [-4.3, 12.52]) + Centroid1 (0.93 [-0.17, 2.02]) + Centroid2 (0.17 [-1.07, 1.41]) + Centroid3 (3.96 [0.63, 7.29]) + BoundingBox1 (1.89 [-3.88, 7.65]) + BoundingBox2 (-0.25 [-7.27, 6.76]) + BoundingBox3 (-2.8 [-7.35, 1.74]) + BoundingBoxVolume (11.53 [1.29, 21.77]) + EquivDiameter (17.86 [2.14, 33.58]) + Extent (4.52 [-1.33, 10.37]) + PrincipalAxisLength1 (-1.71 [-5.2, 1.79]) + PrincipalAxisLength2 (-3.2 [-6.85, 0.45]) + PrincipalAxisLength3 (-1.54 [-3.84, 0.76]) + ConvexVolume (-21.02 [-38.81, -3.23]) + Solidity (-9.3 [-18.06, -0.53]) + SurfaceArea (-3.45 [-8.18, 1.28]) + **age (5.1 [0.76, 9.43])** + gender (1.75 [-1.76, 5.26]) + weight (3.91 [0.53, 7.28]) + BMI (-5.58 [-10.32, -0.84]) + $HBA1_C$ (1.13 [-1.10, 3.36]) + **years.of.diabetes (-1.78 [-3.36, -0.2])** + diagnosisT2D (2.18 [-1.35, 5.72]) + diagnosispreDM (0.78 [-1.48, 3.03])	5.474
		AIRarg ~ BP_{ND} (0.25 [-0.15, 0.65], freq=0.62) + age (0.41 [0.01, 0.81], freq=0.63) + year.of.diabetes (-0.61 [-1.04, -0.18], freq=0.73)	1.946
	Head	AIRarg ~ BP_{ND} (0.34 [-1.31, 1.98]) + Volume (-7.46 [-35.98, 21.07]) + Centroid1 (-0.35 [-1.82, 1.12]) + Centroid2 (0.01 [-1.96, 1.97]) + Centroid3 (-0.63 [-2.95, 1.68]) + BoundingBox1 (-2.18 [-10.47, 6.12]) + BoundingBox2 (-1.04 [-12.1, 10.03]) + BoundingBox3 (-0.56 [-9.9, 8.77]) + BoundingBoxVolume (-1.58 [-29.53, 26.37]) + EquivDiameter (-2.33 [-24.15, 19.48]) + Extent (-2.13 [-12.93, 8.68]) + PrincipalAxisLength1 (0.87 [-3.86, 5.6]) + PrincipalAxisLength2 (0.23 [-5.28, 5.74]) + PrincipalAxisLength3 (-0.42 [-5.49, 4.66]) + ConvexVolume (13.37 [-18.73, 45.46]) + Solidity (3.04 [-8.77, 14.85]) + SurfaceArea (-0.31 [-5.25, 4.63]) + **age (-0.28 [-3.31, 2.76])** + gender (-1.96 [-6.2, 2.27]) + weight (-0.17 [-3.3, 2.96]) + BMI (0.36 [-3.05, 3.76]) + $HBA1_C$ (-1.33 [-5.27, 2.60]) + **years.of.diabetes (-0.76 [-2.71, 1.19])** + diagnosisT2D (2.24 [-4.3, 8.78]) + diagnosispreDM (0.95 [-3.08, 4.98])	10.656
		AIRarg ~ age (0.42 [0.02, 0.83], freq=0.62) + years.of.diabetes (-0.69 [-1.10, -0.29], freq=0.75)	1.974
	Body	AIRarg ~ **BP_{ND} (0.04 [-1.17, 1.26])** + Volume (-1.07 [-12.08, 9.94]) + Centroid1 (0.98 [-0.86, 2.82]) + **Centroid2 (-0.82 [-2.61, 0.96])** + Centroid3 (-0.39 [-1.43, 0.64]) + BoundingBox1 (2.89 [-11.78, 17.56]) + BoundingBox2 (2.38 [-11.62, 16.38]) + **BoundingBox3 (1.89 [-10.44, 14.21])** + BoundingBoxVolume (2.81 [-11.12, 16.75]) + EquivDiameter (-0.82 [-19.41, 17.78]) + Extent (3.7 [-5.38, 12.77]) + PrincipalAxisLength1 (-	9.709

		0.49 [-8.72, 7.74]) + PrincipalAxisLength2 (0.81 [-3, 4.62]) + **PrincipalAxisLength3 (0.88 [-3.92, 5.67])** + ConvexVolume (-5.71 [-21.56, 10.14]) + **Solidity (-1.9 [-6.45, 2.66])** + SurfaceArea (-1.08 [-3.08, 0.91]) + **age (-0.8 [-2.65, 1.06])** + gender (-1.33 [-4.43, 1.77]) + weight (-0.26 [-2.13, 1.62]) + **BMI (0.59 [-1.21, 2.39])** + $HBA1_C$ (-1.3 [-4.19, 1.6]) + **years.of.diabetes (0.27 [-2.29, 2.84])** + diagnosisT2D (0.48 [-4.63, 5.59]) + diagnosispreDM (1.59 [-1.91, 5.09])	
		AIRarg ~ BP_{ND} (0.09 [-0.35, 0.53], freq=0.60) + Centroid2 (-0.09 [-0.54, 0.36], freq=0.52) + BoundingBox3 (0.32 [-0.17, 0.8], freq=0.53) + PrincipalAxisLength3 (0.23 [-0.31, 0.77], freq=0.69) + Solidity (-0.08 [-0.51, 0.35], freq=0.62) + age (0.47 [0.04, 0.91], freq=0.59) + BMI (0.24 [-0.16, 0.63], freq=0.54) + years.of.diabetes (-0.57 [-1.02, -0.11], freq=0.75)	2.079
	Tail	AIRarg ~ **BP_{ND} (0.05 [-0.88, 0.98])** + Volume (2.46 [-5.08, 9.99]) + Centroid1 (-0.67 [-2.03, 0.7]) + Centroid2 (0.54 [-1.16, 2.25]) + Centroid3 (-0.82 [-2.4, 0.76]) + **BoundingBox1 (4.33 [-0.06, 8.73])** + BoundingBox2 (2.73 [-1.37, 6.84]) + BoundingBox3 (0.86 [-4.43, 6.16]) + BoundingBoxVolume (-0.94 [-11.37, 9.5]) + EquivDiameter (-0.95 [-8.72, 6.82]) + Extent (0.23 [-6.2, 6.67]) + PrincipalAxisLength1 (-4.19 [-8.81, 0.42]) + PrincipalAxisLength2 (-1.65 [-4.23, 0.94]) + PrincipalAxisLength3 (-0.7 [-4.23, 2.84]) + ConvexVolume (-1.03 [-16.84, 14.77]) + Solidity (-0.62 [-6.49, 5.25]) + SurfaceArea (-1.03 [-2.15, 0.09]) + **age (0.04 [-0.81, 0.89])** + gender (-1.23 [-3.4, 0.95]) + weight (0.35 [-0.69, 1.38]) + **BMI (0.35 [-0.59, 1.28])** + $HBA1_C$ (0 [-1.64, 1.64]) + **years.of.diabetes (-0.04 [-0.97, 0.88])** + diagnosisT2D (-0.58 [-3.53, 2.36]) + diagnosispreDM (0.81 [-1.36, 2.98])	5.673
		AIRarg ~ BP_{ND} (0.33 [-0.07, 0.73], freq=0.75) + BoundingBox1 (0.3 [-0.12, 0.71], freq=0.64) + age (0.49 [0.08, 0.90], freq=0.7) + BMI (0.12 [-0.26, 0.5], freq=0.58) + years.of.diabetes (-0.55 [-0.98, -0.12], freq=0.77)	2.050
BP_{ND} x Volume	Whole	AIRarg ~ **BP_{ND} x Volume (-0.03 [-0.97, 0.92])** + Volume (-0.85 [-7.29, 5.59]) + **Centroid1 (0.39 [-0.39, 1.18])** + Centroid2 (-0.18 [-1.14, 0.78]) + Centroid3 (0.56 [-0.26, 1.38]) + BoundingBox1 (-1.07 [-5.12, 2.97]) + BoundingBox2 (-2.36 [-7.65, 2.92]) + BoundingBox3 (-1.73 [-5.52, 2.05]) + BoundingBoxVolume (3.78 [-3.24, 10.81]) + EquivDiameter (5.02 [-4.71, 14.75]) + Extent (-0.19 [-3.41, 3.04]) + PrincipalAxisLength1 (0.35 [-2.07, 2.78]) + PrincipalAxisLength2 (-0.16 [-1.98, 1.66]) + PrincipalAxisLength3 (0 [-1.41, 1.42]) + ConvexVolume (-3.84 [-11.92, 4.25]) + Solidity (-1.46 [-6.27, 3.35]) + SurfaceArea (-0.12 [-3.14, 2.91]) + **age (0.92 [-0.12, 1.97])** + gender (-0.62 [-2.67, 1.43]) + weight (0.63 [-0.74, 2.01]) + BMI (-0.82 [-2.31, 0.68]) + $HBA1_C$ (0.26 [-1.01, 1.53]) + **years.of.diabetes (-0.67 [-1.69, 0.34])** + diagnosisT2D (0.6 [-2.31, 3.51]) + diagnosispreDM (0.36 [-1.66, 2.38])	4.605
		AIRarg ~ BP_{ND} x Volume (0.34 [-0.06, 0.73], freq=0.61) + Centroid1 (0.09 [-0.25, 0.43], freq=0.53) + age (0.55 [0.18, 0.92], freq=0.85) + years.of.diabetes (-0.62 [-1.00, -0.24], freq=0.84)	2.179
	Head	AIRarg ~ **BP_{ND} x Volume (0.24 [-0.61, 1.1])** + Volume (-4.28 [-14.88, 6.31]) + **Centroid1 (-0.47 [-1.3, 0.36])** + Centroid2 (-0.02 [-1.13, 1.09]) + Centroid3 (-0.22 [-1.22, 0.78]) + BoundingBox1 (-2.17 [-7.51, 3.17]) + BoundingBox2 (-1.92 [-8.57, 4.74]) + BoundingBox3 (-1.84 [-6.8, 3.13]) + BoundingBoxVolume (2.26 [-10.78, 15.29]) + EquivDiameter (-0.11 [-10.36, 10.13]) + Extent (-0.53 [-4.17, 3.11]) + PrincipalAxisLength1 (-0.08 [-2.06, 1.9]) + PrincipalAxisLength2 (-0.73 [-3.47, 2.02]) +	4.844

		PrincipalAxisLength3 (-0.93 [-4.03, 2.16]) + ConvexVolume (8.19 [-5.03, 21.41]) + Solidity (1.23 [-3.28, 5.74]) + SurfaceArea (-1.01 [-3.68, 1.67]) + **age (0.57 [-0.50, 1.63])** + gender (-0.53 [-2.16, 1.1]) + weight (0.03 [-1.50, 1.57]) + BMI (0.19 [-1.33, 1.72]) + $HBA1_C$ (-0.39 [-1.69, 0.91]) + **years.of.diabetes (-0.73 [-1.62, 0.16])** + diagnosisT2D (1.15 [-1.61, 3.90]) + diagnosispreDM (1.04 [-1.67, 3.75])	
		AIRarg ~ BP_{ND} x Volume (0.26 [-0.14, 0.66], freq=0.60) + Centroid1 (-0.10 [-0.44, 0.25], freq=0.54) + age (0.56 [0.18, 0.94], freq=0.83) + years.of.diabetes (-0.67 [-1.04, -0.31], freq=0.85)	2.143
	Body	AIRarg ~ **BP_{ND} x Volume (-0.03 [-1.18, 1.11])** + Volume (0.22 [-6.85, 7.30]) + **Centroid1 (0.57 [-0.52, 1.66])** + **Centroid2 (-0.22 [-0.98, 0.55])** + **Centroid3 (0.01 [-0.65, 0.68])** + BoundingBox1 (3.15 [-6.87, 13.16]) + BoundingBox2 (2.46 [-6.85, 11.77]) + BoundingBox3 (1.68 [-6.49, 9.86]) + BoundingBoxVolume (-0.04 [-7.94, 7.86]) + EquivDiameter (-0.49 [-14.47, 13.48]) + Extent (2.03 [-4.06, 8.11]) + PrincipalAxisLength1 (-1.13 [-5.73, 3.47]) + PrincipalAxisLength2 (0.16 [-1.52, 1.84]) + **PrincipalAxisLength3 (0.36 [-2.18, 2.9])** + ConvexVolume (-3.42 [-14.24, 7.4]) + **Solidity (-1.17 [-4.17, 1.82])** + SurfaceArea (-0.91 [-2.39, 0.58]) + **age (0.17 [-0.92, 1.27])** + gender (-0.24 [-2.27, 1.78]) + weight (0.05 [-1.03, 1.13]) + **BMI (0.2 [-0.76, 1.16])** + $HBA1_C$ (-0.29 [-1.71, 1.12]) + **years.of.diabetes (-0.59 [-2.02, 0.84])** + diagnosisT2D (0.55 [-2.36, 3.45]) + diagnosispreDM (1.31 [-0.98, 3.59])	4.986
		AIRarg ~ BP_{ND} x Volume (0.06 [-0.45, 0.57], freq=0.55) + Centroid1 (-0.03 [-0.42, 0.36], freq=0.53) + Centroid2 (-0.01 [-0.41, 0.4], freq=0.53) + Centroid3 (0.25 [-0.19, 0.69], freq=0.52) + PrincipalAxisLength3 (0.45 [-0.04, 0.95], freq=0.73) + Solidity (-0.19 [-0.57, 0.19], freq=0.63) + age (0.52 [0.11, 0.93], freq=0.81) + BMI (0.11 [-0.28, 0.5], freq=0.52) + years.of.diabetes (-0.54 [-0.96, -0.11], freq=0.83)	2.550
	Tail	AIRarg ~ **BP_{ND} x Volume (0.14 [-0.73, 1.01])** + Volume (0.6 [-4.75, 5.95]) + **Centroid1 (0.03 [-0.68, 0.74])** + **Centroid2 (0.24 [-0.58, 1.06])** + **Centroid3 (-0.01 [-0.82, 0.80])** + **BoundingBox1 (2.08 [-1.7, 5.87])** + BoundingBox2 (1.41 [-2.34, 5.15]) + BoundingBox3 (0.46 [-3.17, 4.09]) + BoundingBoxVolume (0.37 [-5.91, 6.66]) + EquivDiameter (-1.34 [-8.42, 5.73]) + Extent (0.22 [-3.05, 3.49]) + PrincipalAxisLength1 (-0.96 [-3.62, 1.69]) + PrincipalAxisLength2 (-0.07 [-1.96, 1.82]) + **PrincipalAxisLength3 (0.89 [-1.78, 3.57])** + ConvexVolume (-0.89 [-9.34, 7.57]) + Solidity (0.60 [-2.43, 3.63]) + SurfaceArea (-1.17 [-2.25, -0.10]) + **age (0.6 [-0.17, 1.36])** + gender (-0.44 [-2.00, 1.11]) + weight (0.19 [-0.67, 1.04]) + **BMI (0.08 [-0.72, 0.89])** + $HBA1_C$ (-0.09 [-1.12, 0.94]) + **years.of.diabetes (-0.58 [-1.35, 0.2])** + diagnosisT2D (0.59 [-1.22, 2.39]) + diagnosispreDM (0.84 [-0.76, 2.44])	4.422
		AIRarg ~ BP_{ND} x Volume (0.19 [-0.27, 0.65], freq=0.59) + Centroid1 (0.09 [-0.39, 0.56], freq=0.54) + Centroid2 (-0.08 [-0.51, 0.34], freq=0.50) + Centroid3 (0.15 [-0.31, 0.61], freq=0.52) + BoundingBox1 (0.14 [-0.29, 0.57], freq=0.64) + PrincipalAxisLength3 (0.24 [-0.20, 0.67], freq=0.56) + age (0.6 [0.21, 1.00], freq=0.9) + BMI (0.14 [-0.27, 0.56], freq=0.57) + years.of.diabetes (-0.64 [-1.02, -0.27], freq=0.86)	2.298

Supplementary Table 3 Fitted full and reduced models for predicting primary functional beta cell mass outcome (AIRargMAX) with the primary PET outcome measures (SUVR-1 x Volume, BPnd, and BPnd x Volume), MRI morphology metrics and clinical covariates. Values in parentheses represent coefficient estimates; values in brackets denote 95% confidence intervals for the linear regression models; and "freq = " indicates the selection frequencies of variables retained in each reduced model.

PET outcome measure	Pancreas ROI	Linear Models to Predict maximum insulin response to arginine (AIRargMAX)	CV MSE
SUVR-1 x Volume	Whole	AIRargMAX ~ **SUVR-1 x Volume (0.08 [-2.72, 2.87])** + Volume (3.74 [-12.24, 19.72]) + **Centroid1 (0.06 [-1.45, 1.57])** + **Centroid2 (-1.98 [-3.86, -0.1])** + **Centroid3 (1.44 [-0.15, 3.02])** + **BoundingBox1 (-5.8 [-13.6, 2.01])** + BoundingBox2 (-4.7 [-14.55, 5.15]) + BoundingBox3 (-3.94 [-11.2, 3.33]) + BoundingBoxVolume (1.45 [-11.76, 14.67]) + EquivDiameter (0.43 [-18.05, 18.92]) + Extent (-3.88 [-10.26, 2.5]) + PrincipalAxisLength1 (2.96 [-1.8, 7.71]) + PrincipalAxisLength2 (-0.17 [-3.65, 3.32]) + **PrincipalAxisLength3 (0.31 [-2.42, 3.05])** + ConvexVolume (0.51 [-15.96, 16.98]) + Solidity (0.63 [-8.71, 9.97]) + SurfaceArea (1.77 [-4.09, 7.62]) + **age (1.4 [-0.76, 3.56])** + gender (-3.03 [-6.93, 0.87]) + weight (1.04 [-1.83, 3.9]) + **BMI (-1.48 [-4.81, 1.86])** + **HBA1c (-1.73 [-4.19, 0.72])** + **years.of.diabetes (-1.13 [-3.18, 0.92])** + diagnosisT2D (0.24 [-5.41, 5.89]) + diagnosispreDM (-2.10 [-5.94, 1.75])	9.742
SUVR-1 x Volume	Whole	AIRargMAX ~ SUVR-1 x Volume (1.01 [0.04, 1.99], freq=0.77) + Centroid1 (-0.15 [-1, 0.7], freq=0.55) + Centroid2 (-1.02 [-1.97, -0.06], freq=0.78) + Centroid3 (1.29 [0.36, 2.22], freq=0.85) + BoundingBox1 (-0.45 [-1.35, 0.44], freq=0.52) + PrincipalAxisLength3 (0.48 [-0.42, 1.38], freq=0.6) + age (0.7 [-0.18, 1.58], freq=0.63) + BMI (-0.77 [-1.63, 0.08], freq=0.58) + HBA1c (-1.80 [-2.96, -0.63], freq=0.97) + years.of.diabetes (-1.23 [-2.52, 0.06], freq=0.87)	5.885
SUVR-1 x Volume	Head	AIRargMAX ~ **SUVR-1 x Volume (0.08 [-2.86, 3.02])** + Volume (-2.39 [-23.31, 18.53]) + **Centroid1 (-0.6 [-2.19, 0.98])** + **Centroid2 (-0.83 [-3.19, 1.53])** + **Centroid3 (0.66 [-1.96, 3.28])** + **BoundingBox1 (-8.87 [-18.7, 0.97])** + BoundingBox2 (-9.58 [-22.02, 2.85]) + BoundingBox3 (-8.12 [-17.41, 1.16]) + BoundingBoxVolume (10.59 [-15.67, 36.84]) + EquivDiameter (13.12 [-9.69, 35.94]) + Extent (-2.91 [-12.5, 6.68]) + PrincipalAxisLength1 (-1.13 [-5.09, 2.84]) + **PrincipalAxisLength2 (-3.84 [-9.38, 1.71])** + **PrincipalAxisLength3 (-3.26 [-9.25, 2.73])** + ConvexVolume (5.89 [-19.76, 31.54]) + Solidity (-2.33 [-11.19, 6.54]) + SurfaceArea (-1.81 [-6.93, 3.31]) + **age (1.15 [-1.71, 4.01])** + gender (-0.59 [-3.68, 2.51]) + weight (0.37 [-3.15, 3.88]) + BMI (-0.71 [-4.32, 2.91]) + **HBA1c (-1.07 [-3.54, 1.4])** + **years.of.diabetes (-1.22 [-3.14, 0.70])** + diagnosisT2D (-1.61 [-6.89, 3.67]) + diagnosispreDM (0.86 [-4.64, 6.37])	9.345
SUVR-1 x Volume	Head	AIRargMAX ~ SUVR-1 x Volume (1.1 [-0.02, 2.22], freq=0.73) + Centroid1 (-0.57 [-1.55, 0.41], freq=0.60) + Centroid2 (-1.02 [-2.01, -0.02], freq=0.84) + Centroid3 (0.55 [-0.33, 1.42], freq=0.71) + BoundingBox1 (-0.54 [-1.53, 0.46], freq=0.52) + PrincipalAxisLength2 (-1.16 [-2.28, -0.03], freq=0.91) + PrincipalAxisLength3 (0.47 [-0.51, 1.45], freq=0.61) + age (0.66 [-0.24, 1.56], freq=0.65) + HBA1c (-1.04 [-2.28, 0.20], freq=0.89) + years.of.diabetes (-1.51 [-2.71, -0.31], freq=0.91)	5.539

	Body	AIRargMAX ~ **SUVR-1 x Volume (-0.21 [-2.95, 2.54])** + Volume (10.97 [-5.89, 27.83]) + **Centroid1 (-0.11 [-2.44, 2.22])** + **Centroid2 (-0.39 [-2.05, 1.26])** + **Centroid3 (1.05 [-0.41, 2.5])** + BoundingBox1 (14.71 [-4.78, 34.2]) + BoundingBox2 (13.23 [-5.33, 31.79]) + BoundingBox3 (9.9 [-6.77, 26.56]) + BoundingBoxVolume (-8.5 [-25.62, 8.61]) + EquivDiameter (-15.13 [-43.63, 13.38]) + Extent (7.36 [-4.65, 19.38]) + PrincipalAxisLength1 (-7.26 [-16.48, 1.95]) + PrincipalAxisLength2 (-1.22 [-4.78, 2.33]) + **PrincipalAxisLength3 (-1.95 [-7.4, 3.5])** + ConvexVolume (-3.11 [-24.14, 17.92]) + Solidity (-1.78 [-8, 4.43]) + SurfaceArea (-1.02 [-3.98, 1.94]) + **age (0.69 [-1.39, 2.78])** + gender (-1.43 [-6.07, 3.21]) + weight (0.05 [-2.24, 2.34]) + BMI (-0.19 [-2.25, 1.87]) + **HBA1$_{C}$ (-0.94 [-4.10, 2.22])** + **years.of.diabetes (-0.11 [-3.22, 2.99])** + diagnosisT2D (-5.41 [-11.21, 0.40]) + diagnosispreDM (-0.69 [-5.60, 4.22])	11.386
		AIRargMAX ~ SUVR-1 x Volume (0.45 [-0.75, 1.65], freq=0.61) + Centroid1 (-0.45 [-1.31, 0.41], freq=0.57) + Centroid2 (-0.79 [-1.64, 0.06], freq=0.78) + Centroid3 (1.41 [0.55, 2.27], freq=0.83) + PrincipalAxisLength3 (1.34 [0.25, 2.44], freq=0.83) + age (0.99 [0.14, 1.85], freq=0.71) + HBA1$_{C}$ (-1.49 [-2.61, -0.38], freq=0.93) + years.of.diabetes (-1.15 [-2.35, 0.05], freq=0.84)	5.378
	Tail	AIRargMAX ~ **SUVR-1 x Volume (1.68 [-2.24, 5.6])** + Volume (1.01 [-16.92, 18.94]) + **Centroid1 (0.25 [-1.68, 2.17])** + **Centroid2 (-0.95 [-3.42, 1.52])** + **Centroid3 (1.67 [-0.57, 3.92])** + BoundingBox1 (2 [-9.51, 13.52]) + BoundingBox2 (0.94 [-10.19, 12.08]) + BoundingBox3 (2.99 [-7.69, 13.67]) + BoundingBoxVolume (2.24 [-16.32, 20.79]) + EquivDiameter (-4.13 [-23.27, 15.01]) + Extent (2.09 [-6.9, 11.09]) + PrincipalAxisLength1 (0.18 [-7.5, 7.87]) + PrincipalAxisLength2 (1.07 [-4.10, 6.23]) + **PrincipalAxisLength3 (0.83 [-6.2, 7.87])** + ConvexVolume (-3.36 [-28.29, 21.58]) + Solidity (-0.04 [-8.29, 8.2]) + SurfaceArea (-1.54 [-4.55, 1.48]) + **age (1.02 [-1.06, 3.09])** + gender (-0.54 [-4.6, 3.52]) + weight (0.41 [-1.92, 2.74]) + BMI (-0.13 [-2.36, 2.11]) + **HBA1$_{C}$ (-1.16 [-4.01, 1.69])** + **years.of.diabetes (-0.87 [-3.11, 1.37])** + diagnosisT2D (-3.16 [-8.15, 1.83]) + diagnosispreDM (-1.98 [-6.13, 2.17])	11.472
		AIRargMAX ~ SUVR-1 x Volume (0.63 [-0.51, 1.78], freq=0.64) + Centroid1 (-0.37 [-1.42, 0.67], freq=0.64) + Centroid2 (-0.61 [-1.68, 0.46], freq=0.6) + Centroid3 (0.76 [-0.26, 1.78], freq=0.75) + PrincipalAxisLength3 (0.34 [-0.72, 1.4], freq=0.50) + age (0.71 [-0.25, 1.68], freq=0.62) + HBA1$_{C}$ (-1.92 [-3.15, -0.69], freq=0.94) + years.of.diabetes (-1.47 [-2.81, -0.12], freq=0.90)	5.532
BP_{ND}	Whole	AIRargMAX ~ **BP_{ND} (-1.53 [-6.79, 3.73])** + Volume (6.09 [-14.06, 26.23]) + **Centroid1 (0.63 [-1.98, 3.25])** + **Centroid2 (-1.9 [-4.87, 1.07])** + **Centroid3 (4.43 [-3.55, 12.41])** + **BoundingBox1 (-1.66 [-15.48, 12.15])** + BoundingBox2 (-1.01 [-17.83, 15.8]) + BoundingBox3 (-3.14 [-14.02, 7.74]) + BoundingBoxVolume (7.32 [-17.22, 31.86]) + EquivDiameter (12.13 [-25.54, 49.8]) + Extent (1.07 [-12.96, 15.09]) + PrincipalAxisLength1 (1.16 [-7.22, 9.54]) + PrincipalAxisLength2 (-1.8 [-10.55, 6.95]) + **PrincipalAxisLength3 (-0.21 [-5.72, 5.3])** + ConvexVolume (-15.31 [-57.92, 27.31]) + Solidity (-5.57 [-26.58, 15.44]) + SurfaceArea (-2.05 [-13.39, 9.29]) + age (4.14 [-6.25, 14.53]) + gender (-0.56 [-8.97, 7.85]) + weight (3.42 [-4.68, 11.52]) + BMI (-5.3 [-16.66, 6.06]) + **HBA1$_{C}$ (-2.27 [-7.61, 3.08])** + **years.of.diabetes (-1.93 [-5.72, 1.85])** + diagnosisT2D (2.65 [-5.82, 11.12]) + diagnosispreDM (-1.58 [-6.99, 3.82])	14.834

		AIRargMAX ~ BP_{ND} (0.86 [-0.12, 1.85], freq=0.79) + Centroid1 (-0.24 [-1.22, 0.74], freq=0.51) + Centroid2 (-1.25 [-2.34, -0.15], freq=0.8) + Centroid3 (1.11 [0.18, 2.03], freq=0.88) + BoundingBox1 (-0.77 [-1.88, 0.34], freq=0.5) + PrincipalAxisLength3 (0.9 [0, 1.8], freq=0.61) + $HBA1_C$ (-2.62 [-3.84, -1.41], freq=0.97) + years.of.diabetes (-0.85 [-2.14, 0.45], freq=0.78)	5.073
	Head	AIRargMAX ~ **BP_{ND} (0.81 [-1.92, 3.54])** + Volume (-6.3 [-53.61, 41.01]) + Centroid1 (-0.5 [-2.94, 1.94]) + **Centroid2 (-1.89 [-5.15, 1.38])** + **Centroid3 (0.4 [-3.44, 4.24])** + BoundingBox1 (-8.03 [-21.79, 5.73]) + BoundingBox2 (-6.88 [-25.23, 11.47]) + BoundingBox3 (-5.08 [-20.56, 10.41]) + BoundingBoxVolume (4.22 [-42.14, 50.58]) + EquivDiameter (2.75 [-33.44, 38.94]) + Extent (-4.93 [-22.85, 12.99]) + PrincipalAxisLength1 (1.13 [-6.71, 8.97]) + **PrincipalAxisLength2 (-1.05 [-10.19, 8.09])** + **PrincipalAxisLength3 (-1.26 [-9.67, 7.16])** + ConvexVolume (16.16 [-37.08, 69.4]) + Solidity (2.5 [-17.08, 22.09]) + SurfaceArea (-1.22 [-9.42, 6.97]) + **age (-0.06 [-5.1, 4.97])** + gender (-3.04 [-10.07, 3.98]) + weight (0.4 [-4.79, 5.58]) + BMI (-0.89 [-6.54, 4.75]) + **$HBA1_C$ (-4.08 [-10.6, 2.45])** + **years.of.diabetes (-1.4 [-4.64, 1.83])** + diagnosisT2D (3.50 [-7.34, 14.35]) + diagnosispreDM (0.03 [-6.64, 6.71])	12.820
		AIRargMAX ~ BP_{ND} (0.81 [-0.18, 1.79], freq=0.75) + Centroid2 (-1.25 [-2.28, -0.22], freq=0.81) + Centroid3 (0.69 [-0.26, 1.64], freq=0.69) + PrincipalAxisLength2 (-0.97 [-2.07, 0.12], freq=0.81) + PrincipalAxisLength3 (0.91 [0.06, 1.76], freq=0.59) + age (0.28 [-0.74, 1.31], freq=0.54) + $HBA1_C$ (-1.47 [-2.94, -0.01], freq=0.81) + years.of.diabetes (-1.28 [-2.59, 0.04], freq=0.87)	5.021
	Body	AIRargMAX ~ **BP_{ND} (-0.44 [-2.91, 2.04])** + Volume (4.74 [-17.68, 27.15]) + **Centroid1 (-0.85 [-4.6, 2.89])** + **Centroid2 (-1.56 [-5.21, 2.08])** + **Centroid3 (1.23 [-0.88, 3.34])** + BoundingBox1 (17.08 [-12.77, 46.94]) + BoundingBox2 (15.02 [-13.47, 43.51]) + BoundingBox3 (11.6 [-13.48, 36.68]) + BoundingBoxVolume (-8.44 [-36.8, 19.91]) + EquivDiameter (-21.63 [-59.47, 16.21]) + Extent (8.86 [-9.6, 27.33]) + PrincipalAxisLength1 (-5.62 [-22.36, 11.12]) + **PrincipalAxisLength2 (0.27 [-7.49, 8.03])** + **PrincipalAxisLength3 (-0.31 [-10.06, 9.45])** + ConvexVolume (4.57 [-27.69, 36.83]) + Solidity (1.18 [-8.1, 10.46]) + SurfaceArea (-2.33 [-6.4, 1.73]) + **age (-0.03 [-3.81, 3.75])** + gender (-0.99 [-7.3, 5.31]) + weight (-1.21 [-5.02, 2.6]) + BMI (0.9 [-2.76, 4.55]) + **$HBA1_C$ (-2.02 [-7.91, 3.88])** + **years.of.diabetes (0.44 [-4.78, 5.66])** + diagnosisT2D (-5.04 [-15.44, 5.35]) + diagnosispreDM (-2.01 [-9.13, 5.11])	23.919
		AIRargMAX ~ BP_{ND} (0.24 [-0.71, 1.20], freq=0.66) + Centroid1 (-0.45 [-1.34, 0.44], freq=0.56) + Centroid2 (-1.07 [-1.98, -0.17], freq=0.81) + Centroid3 (1.71 [0.78, 2.64], freq=0.85) + PrincipalAxisLength2 (0.66 [-0.36, 1.67], freq=0.54) + PrincipalAxisLength3 (1.35 [0.25, 2.44], freq=0.78) + age (0.74 [-0.25, 1.73], freq=0.57) + $HBA1_C$ (-2.44 [-3.73, -1.14], freq=0.97) + years.of.diabetes (-0.35 [-1.69, 0.99], freq=0.72)	4.793
	Tail	AIRargMAX ~ **BP_{ND} (-0.20 [-3.67, 3.27])** + Volume (3.88 [-24.25, 32.01]) + **Centroid1 (-2.68 [-7.78, 2.41])** + **Centroid2 (2.21 [-4.14, 8.57])** + **Centroid3 (-1.61 [-7.5, 4.28])** + BoundingBox1 (4.39 [-12.02, 20.79]) + BoundingBox2 (2.18 [-13.14, 17.5]) + BoundingBox3 (-6.67 [-26.41, 13.08]) + BoundingBoxVolume (-16.3 [-55.23, 22.64]) + EquivDiameter (-8.07 [-37.06, 20.93]) + Extent (-10.95 [-34.95, 13.05]) + PrincipalAxisLength1 (-7.52 [-24.76, 9.72]) + PrincipalAxisLength2 (-0.97 [-10.61, 8.68]) +	59.387

		**PrincipalAxisLength3 (4.36 [-8.84, 17.56])** + ConvexVolume (21.63 [-37.34, 80.61]) + Solidity (7.37 [-14.55, 29.28]) + SurfaceArea (-0.9 [-5.07, 3.27]) + age (0.39 [-2.79, 3.56]) + gender (-3.41 [-11.53, 4.72]) + weight (1.5 [-2.36, 5.37]) + BMI (-0.6 [-4.1, 2.90]) + **$HBA1_C$ (-3.61 [-9.74, 2.51])** + **years.of.diabetes (-0.01 [-3.46, 3.44])** + diagnosisT2D (-0.13 [-11.12, 10.85]) + diagnosispreDM (1.23 [-6.87, 9.33])	
		AIRargMAX ~ BP_{ND} (0.83 [-0.23, 1.88], freq=0.70) + Centroid1 (-0.64 [-1.83, 0.55], freq=0.68) + Centroid2 (-0.91 [-2.12, 0.31], freq=0.52) + Centroid3 (1.03 [-0.16, 2.22], freq=0.71) + PrincipalAxisLength3 (0.48 [-0.43, 1.38], freq=0.6) + $HBA1_C$ (-2.65 [-3.91, -1.39], freq=0.93) + years.of.diabetes (-0.91 [-2.24, 0.43], freq=0.82)	5.434
BP_{ND} x Volume	Whole	AIRargMAX ~ **BP_{ND} x Volume (0.28 [-1.55, 2.1])** + Volume (3.24 [-9.17, 15.65]) + **Centroid1 (0.02 [-1.49, 1.54])** + **Centroid2 (-1.96 [-3.82, -0.11])** + **Centroid3 (1.41 [-0.16, 2.99])** + **BoundingBox1 (-5.35 [-13.15, 2.44])** + BoundingBox2 (-4.13 [-14.32, 6.06]) + BoundingBox3 (-3.48 [-10.79, 3.82]) + BoundingBoxVolume (0.74 [-12.8, 14.28]) + EquivDiameter (-0.1 [-18.86, 18.65]) + Extent (-3.75 [-9.97, 2.47]) + PrincipalAxisLength1 (2.93 [-1.74, 7.6]) + PrincipalAxisLength2 (-0.06 [-3.57, 3.46]) + **PrincipalAxisLength3 (0.36 [-2.37, 3.09])** + ConvexVolume (1.08 [-14.5, 16.66]) + Solidity (1.06 [-8.21, 10.33]) + SurfaceArea (1.75 [-4.08, 7.58]) + **age (1.4 [-0.62, 3.42])** + gender (-2.86 [-6.81, 1.08]) + weight (0.91 [-1.74, 3.56]) + **BMI (-1.32 [-4.2, 1.57])** + **$HBA1_C$ (-1.71 [-4.16, 0.73])** + **years.of.diabetes (-1.12 [-3.08, 0.84])** + diagnosisT2D (0.14 [-5.46, 5.74]) + diagnosispreDM (-2.22 [-6.11, 1.67])	9.906
		AIRargMAX ~ BP_{ND} x Volume (1.03 [0.06, 2], freq=0.81) + Centroid1 (-0.06 [-0.89, 0.78], freq=0.55) + Centroid2 (-0.88 [-1.81, 0.06], freq=0.77) + Centroid3 (1.21 [0.28, 2.13], freq=0.84) + BoundingBox1 (-0.5 [-1.39, 0.4], freq=0.53) + PrincipalAxisLength3 (0.51 [-0.38, 1.4], freq=0.6) + age (0.82 [-0.08, 1.73], freq=0.67) + BMI (-0.78 [-1.63, 0.07], freq=0.57) + $HBA1_C$ (-1.72 [-2.89, -0.55], freq=0.97) + years.of.diabetes (-1.27 [-2.55, 0.02], freq=0.86)	5.833
	Head	AIRargMAX ~ **BP_{ND} x Volume (0.81 [-0.76, 2.37])** + Volume (1.15 [-18.22, 20.52]) + **Centroid1 (-0.62 [-2.13, 0.9])** + **Centroid2 (-0.51 [-2.54, 1.52])** + **Centroid3 (0.24 [-1.59, 2.07])** + **BoundingBox1 (-10.23 [-19.99, -0.47])** + BoundingBox2 (-10.96 [-23.12, 1.21]) + BoundingBox3 (-9.12 [-18.19, -0.04]) + BoundingBoxVolume (15.47 [-8.35, 39.29]) + EquivDiameter (11.93 [-6.79, 30.65]) + Extent (-1.59 [-8.24, 5.07]) + PrincipalAxisLength1 (-1.15 [-4.77, 2.47]) + **PrincipalAxisLength2 (-4.18 [-9.2, 0.84])** + **PrincipalAxisLength3 (-3.06 [-8.71, 2.6])** + ConvexVolume (1.05 [-23.1, 25.2]) + Solidity (-3.92 [-12.16, 4.32]) + SurfaceArea (-1.73 [-6.61, 3.15]) + **age (0.91 [-1.04, 2.86])** + gender (-0.28 [-3.25, 2.7]) + weight (-0.12 [-2.93, 2.68]) + BMI (-0.24 [-3.03, 2.55]) + **$HBA1_C$ (-0.9 [-3.28, 1.48])** + **years.of.diabetes (-1.22 [-2.84, 0.4])** + diagnosisT2D (-1.53 [-6.56, 3.5]) + diagnosispreDM (1.23 [-3.73, 6.18])	8.828
		AIRargMAX ~ BP_{ND} x Volume (1.02 [-0.05, 2.08], freq=0.79) + Centroid1 (-0.54 [-1.53, 0.44], freq=0.59) + Centroid2 (-0.92 [-1.91, 0.08], freq=0.85) + Centroid3 (0.57 [-0.3, 1.44], freq=0.71) + BoundingBox1 (-0.66 [-1.66, 0.35], freq=0.56) + PrincipalAxisLength2 (-0.9 [-1.94, 0.14], freq=0.9) + PrincipalAxisLength3 (0.57 [-0.38, 1.52], freq=0.59) + age (0.86 [-0.08, 1.8], freq=0.69) + $HBA1_C$ (-1.06 [-2.3, 0.18], freq=0.88) + years.of.diabetes (-1.55 [-2.75, -0.36], freq=0.90)	5.452

	Body	AIRargMAX ~ ***BP_{ND}* x Volume (-0.85 [-3.24, 1.54])** + Volume (10.79 [-3.99, 25.58]) + **Centroid1 (-0.08 [-2.36, 2.2])** + **Centroid2 (-0.51 [-2.11, 1.1])** + **Centroid3 (1.03 [-0.37, 2.42])** + BoundingBox1 (18.7 [-2.24, 39.64]) + BoundingBox2 (16.95 [-2.52, 36.42]) + BoundingBox3 (13.08 [-4.01, 30.17]) + BoundingBoxVolume (-11.45 [-27.96, 5.07]) + EquivDiameter (-20.31 [-49.53, 8.91]) + Extent (9.44 [-3.28, 22.16]) + PrincipalAxisLength1 (-8.64 [-18.26, 0.99]) + PrincipalAxisLength2 (-1.73 [-5.24, 1.79]) + **PrincipalAxisLength3 (-2.51 [-7.82, 2.79])** + ConvexVolume (0.46 [-22.16, 23.09]) + Solidity (-1.21 [-7.47, 5.06]) + SurfaceArea (-1.44 [-4.54, 1.67]) + **age (0.3 [-1.99, 2.59])** + gender (-1.59 [-5.83, 2.64]) + weight (-0.06 [-2.32, 2.2]) + BMI (-0.27 [-2.27, 1.73]) + **$HBA1_C$ (-0.77 [-3.73, 2.2])** + **years.of.diabetes (-0.01 [-3, 2.99])** + diagnosisT2D (-6.42 [-12.5, -0.34]) + diagnosispreDM (-0.49 [-5.27, 4.30])	12.074
		AIRargMAX ~ BP_{ND} x Volume (0.46 [-0.61, 1.53], freq=0.66) + Centroid1 (-0.38 [-1.18, 0.41], freq=0.55) + Centroid2 (-0.73 [-1.58, 0.11], freq=0.77) + Centroid3 (1.37 [0.51, 2.23], freq=0.82) + PrincipalAxisLength3 (1.37 [0.33, 2.4], freq=0.83) + age (1.06 [0.18, 1.94], freq=0.72) + $HBA1_C$ (-1.47 [-2.58, -0.35], freq=0.93) + years.of.diabetes (-1.17 [-2.36, 0.02], freq=0.84)	5.327
	Tail	AIRargMAX ~ ***BP_{ND}* x Volume (1.13 [-1.21, 3.47])** + Volume (2.98 [-11.41, 17.37]) + **Centroid1 (0.23 [-1.68, 2.13])** + **Centroid2 (-0.5 [-2.71, 1.72])** + **Centroid3 (1.5 [-0.68, 3.68])** + BoundingBox1 (0.2 [-9.99, 10.38]) + BoundingBox2 (-0.6 [-10.67, 9.47]) + BoundingBox3 (1.35 [-8.41, 11.1]) + BoundingBoxVolume (3.91 [-13, 20.81]) + EquivDiameter (-4.45 [-23.48, 14.58]) + Extent (1.03 [-7.76, 9.82]) + PrincipalAxisLength1 (1.45 [-5.69, 8.6]) + PrincipalAxisLength2 (1.92 [-3.16, 7.00]) + **PrincipalAxisLength3 (2.15 [-5.05, 9.34])** + ConvexVolume (-5.03 [-27.76, 17.70]) + Solidity (0.03 [-8.12, 8.17]) + SurfaceArea (-1.68 [-4.58, 1.22]) + **age (1.14 [-0.93, 3.21])** + gender (-0.03 [-4.22, 4.16]) + weight (0.29 [-2.02, 2.59]) + BMI (-0.24 [-2.42, 1.93]) + **$HBA1_C$ (-1.2 [-3.97, 1.57])** + **years.of.diabetes (-1.14 [-3.23, 0.95])** + diagnosisT2D (-2.87 [-7.73, 1.99]) + diagnosispreDM (-1.38 [-5.68, 2.92])	9.744
		AIRargMAX ~ BP_{ND} x Volume (0.79 [-0.23, 1.82], freq=0.81) + Centroid1 (-0.37 [-1.4, 0.65], freq=0.65) + Centroid2 (-0.59 [-1.59, 0.41], freq=0.61) + Centroid3 (0.66 [-0.34, 1.66], freq=0.73) + PrincipalAxisLength3 (0.34 [-0.63, 1.32], freq=0.53) + age (0.81 [-0.15, 1.77], freq=0.65) + $HBA1_C$ (-1.88 [-3.09, -0.67], freq=0.94) + years.of.diabetes (-1.48 [-2.79, -0.18], freq=0.90)	5.501

Supplementary Table 4 Fitted full and reduced models for predicting primary functional beta cell mass outcome (acute:MAX) with the primary PET outcome measures (SUVR-1 x Volume, BPnd, and BPnd x Volume), MRI morphology metrics and clinical covariates. Values in parentheses represent coefficient estimates; values in brackets denote 95% confidence intervals for the linear regression models; and "freq = " indicates the selection frequencies of variables retained in each reduced model.

PET outcome measure	Pancreas ROI	Linear Models to Predict ratio of acute to maximum insulin response to arginine (acute:MAX)	CV MSE
SUVR-1 x Volume	Whole	acute:MAX ~ **SUVR-1 x Volume (-0.03 [-0.17, 0.11])** + Volume (0.01 [-0.78, 0.79]) + **Centroid1 (0.05 [-0.02, 0.13])** + **Centroid2 (0.05 [-0.05, 0.14])** + **Centroid3 (0.04 [-0.04, 0.12])** + **BoundingBox1 (0.25 [-0.13,**	0.559

		**0.64])** + BoundingBox2 (0.13 [-0.36, 0.62]) + **BoundingBox3 (0.13 [-0.23, 0.49])** + BoundingBoxVolume (0.28 [-0.37, 0.93]) + EquivDiameter (0.28 [-0.63, 1.19]) + Extent (0.34 [0.02, 0.65]) + **PrincipalAxisLength1 (0.01 [-0.22, 0.25])** + **PrincipalAxisLength2 (0.08 [-0.1, 0.25])** + **PrincipalAxisLength3 (0.01 [-0.12, 0.15])** + ConvexVolume (-0.65 [-1.47, 0.16]) + Solidity (-0.24 [-0.7, 0.22]) + **SurfaceArea (-0.18 [-0.47, 0.1])** + **age (0.07 [-0.04, 0.17])** + gender (0.03 [-0.16, 0.22]) + weight (0.03 [-0.11, 0.17]) + **BMI (-0.06 [-0.22, 0.11])** + **HBA1C (0.24 [0.12, 0.36])** + **years.of.diabetes (-0.1 [-0.2, 0])** + diagnosisT2D (-0.04 [-0.32, 0.24]) + diagnosispreDM (0.06 [-0.13, 0.25])	
		acute:MAX ~ SUVR-1 x Volume (0.02 [-0.05, 0.09], freq=0.53) + Centroid1 (0.03 [-0.03, 0.08], freq=0.77) + Centroid2 (0.01 [-0.05, 0.07], freq=0.59) + Centroid3 (-0.02 [-0.08, 0.04], freq=0.59) + BoundingBox1 (0.05 [-0.06, 0.15], freq=0.57) + BoundingBox3 (0.06 [0.00, 0.12], freq=0.67) + PrincipalAxisLength1 (0.05 [-0.06, 0.16], freq=0.63) + PrincipalAxisLength2 (0.02 [-0.06, 0.1], freq=0.54) + PrincipalAxisLength3 (-0.06 [-0.14, 0.03], freq=0.72) + SurfaceArea (-0.09 [-0.22, 0.03], freq=0.51) + age (0.04 [-0.01, 0.09], freq=0.73) + BMI (0.02 [-0.03, 0.07], freq=0.52) + HBA1C (0.22 [0.15, 0.29], freq=0.98) + years.of.diabetes (-0.12 [-0.2, -0.04], freq=0.69)	0.346
	Head	acute:MAX ~ **SUVR-1 x Volume (-0.02 [-0.25, 0.2])** + Volume (-0.57 [-2.16, 1.02]) + Centroid1 (-0.05 [-0.17, 0.07]) + **Centroid2 (-0.06 [-0.23, 0.12])** + **Centroid3 (0.07 [-0.13, 0.27])** + BoundingBox1 (0.45 [-0.29, 1.2]) + BoundingBox2 (0.49 [-0.45, 1.44]) + BoundingBox3 (0.37 [-0.34, 1.07]) + BoundingBoxVolume (-1.42 [-3.41, 0.58]) + EquivDiameter (-0.14 [-1.87, 1.6]) + Extent (-0.33 [-1.06, 0.40]) + **PrincipalAxisLength1 (0.05 [-0.25, 0.35])** + **PrincipalAxisLength2 (0.2 [-0.22, 0.62])** + **PrincipalAxisLength3 (-0.03 [-0.49, 0.42])** + ConvexVolume (1.16 [-0.79, 3.11]) + Solidity (0.43 [-0.25, 1.1]) + SurfaceArea (-0.18 [-0.57, 0.21]) + **age (0.16 [-0.06, 0.37])** + gender (0 [-0.23, 0.24]) + weight (0.06 [-0.2, 0.33]) + BMI (-0.03 [-0.30, 0.24]) + **HBA1C (0.17 [-0.02, 0.36])** + **years.of.diabetes (-0.13 [-0.28, 0.02])** + diagnosisT2D (0.07 [-0.33, 0.47]) + diagnosispreDM (-0.17 [-0.59, 0.24])	0.694
		acute:MAX ~ SUVR-1 x Volume (0.03 [-0.04, 0.11], freq=0.51) + Centroid2 (0.02 [-0.04, 0.08], freq=0.51) + Centroid3 (-0.01 [-0.06, 0.05], freq=0.51) + PrincipalAxisLength1 (-0.03 [-0.09, 0.02], freq=0.57) + PrincipalAxisLength2 (0.02 [-0.05, 0.09], freq=0.57) + PrincipalAxisLength3 (-0.05 [-0.10, 0.01], freq=0.60) + age (0.05 [0.00, 0.10], freq=0.66) + HBA1C (0.20 [0.12, 0.28], freq=0.96) + years.of.diabetes (-0.1 [-0.18, -0.02], freq=0.60)	0.352
	Body	acute:MAX ~ **SUVR-1 x Volume (0.04 [-0.07, 0.16])** + Volume (-0.5 [-1.22, 0.23]) + **Centroid1 (0.04 [-0.06, 0.14])** + **Centroid2 (-0.06 [-0.13, 0.02])** + **Centroid3 (0.04 [-0.02, 0.11])** + **BoundingBox1 (-0.49 [-1.32, 0.35])** + BoundingBox2 (-0.5 [-1.3, 0.29]) + **BoundingBox3 (-0.35 [-1.07, 0.36])** + BoundingBoxVolume (0.35 [-0.38, 1.09]) + EquivDiameter (0.55 [-0.67, 1.77]) + **Extent (-0.29 [-0.8, 0.23])** + PrincipalAxisLength1 (0.43 [0.03, 0.82]) + **PrincipalAxisLength2 (0.09 [-0.06, 0.24])** + PrincipalAxisLength3 (0.25 [0.02, 0.48]) + ConvexVolume (0 [-0.90, 0.90]) + **Solidity (0.08 [-0.19, 0.35])** + SurfaceArea (-0.12 [-0.24, 0.01]) + **age (0.07 [-0.02, 0.16])** + gender (0.16 [-0.04, 0.36]) + **weight (0 [-0.09, 0.10])** + **BMI (0.01 [-0.08, 0.09])** + **HBA1C (0.21 [0.08, 0.35])** + **years.of.diabetes (-0.19 [-0.32, -0.05])** + diagnosisT2D (0.24 [-0.01, 0.49]) + diagnosispreDM (0.08 [-0.13, 0.29])	0.422

		acute:MAX ~ SUVR-1 x Volume (0.01 [-0.06, 0.08], freq=0.52) + Centroid1 (0.01 [-0.06, 0.07], freq=0.69) + Centroid2 (-0.02 [-0.07, 0.04], freq=0.65) + Centroid3 (0.03 [-0.03, 0.10], freq=0.65) + BoundingBox1 (0.08 [0, 0.15], freq=0.68) + BoundingBox3 (0.02 [-0.05, 0.09], freq=0.6) + Extent (-0.02 [-0.12, 0.07], freq=0.57) + PrincipalAxisLength2 (-0.06 [-0.12, -0.01], freq=0.75) + Solidity (0.03 [-0.05, 0.11], freq=0.58) + age (0.07 [0, 0.15], freq=0.75) + weight (0.03 [-0.06, 0.13], freq=0.55) + BMI (-0.03 [-0.11, 0.06], freq=0.55) + HBA1$_C$ (0.24 [0.17, 0.32], freq=0.98) + years.of.diabetes (-0.11 [-0.20, -0.03], freq=0.71)	0.372
	Tail	acute:MAX ~ SUVR-1 x Volume (-0.04 [-0.24, 0.16]) + Volume (0.15 [-0.77, 1.07]) + **Centroid1 (0.02 [-0.08, 0.12])** + **Centroid2 (0.08 [-0.05, 0.21])** + **Centroid3 (0 [-0.12, 0.11])** + BoundingBox1 (0.41 [-0.18, 1]) + BoundingBox2 (0.42 [-0.15, 0.99]) + BoundingBox3 (0.24 [-0.31, 0.78]) + BoundingBoxVolume (-0.58 [-1.53, 0.37]) + EquivDiameter (-0.64 [-1.62, 0.34]) + Extent (0.01 [-0.45, 0.48]) + PrincipalAxisLength1 (-0.04 [-0.43, 0.36]) + **PrincipalAxisLength2 (0.01 [-0.25, 0.28])** + PrincipalAxisLength3 (0.19 [-0.17, 0.56]) + ConvexVolume (0.27 [-1.01, 1.54]) + Solidity (0.23 [-0.19, 0.65]) + SurfaceArea (-0.04 [-0.2, 0.11]) + **age (0.08 [-0.02, 0.19])** + gender (0 [-0.21, 0.21]) + weight (0.05 [-0.07, 0.17]) + BMI (-0.05 [-0.17, 0.06]) + **HBA1$_C$ (0.21 [0.07, 0.36])** + **years.of.diabetes (-0.13 [-0.25, -0.02])** + diagnosisT2D (0.14 [-0.12, 0.39]) + diagnosispreDM (0.16 [-0.05, 0.38])	0.591
		acute:MAX ~ Centroid1 (0.05 [-0.01, 0.1], freq=0.72) + Centroid2 (0.01 [-0.05, 0.07], freq=0.60) + Centroid3 (0.02 [-0.03, 0.08], freq=0.52) + PrincipalAxisLength2 (-0.02 [-0.07, 0.03], freq=0.63) + age (0.05 [0.00, 0.10], freq=0.70) + HBA1$_C$ (0.21 [0.14, 0.28], freq=0.97) + years.of.diabetes (-0.10 [-0.18, -0.03], freq=0.63)	0.327
BP_{ND}	Whole	acute:MAX ~ **BP_{ND} (-0.15 [-0.39, 0.1])** + Volume (0.26 [-0.66, 1.19]) + **Centroid1 (0.08 [-0.04, 0.2])** + Centroid2 (0.06 [-0.08, 0.2]) + **Centroid3 (0.31 [-0.06, 0.68])** + **BoundingBox1 (0.53 [-0.1, 1.16])** + BoundingBox2 (0.38 [-0.40, 1.15]) + **BoundingBox3 (0.1 [-0.4, 0.60])** + BoundingBoxVolume (0.75 [-0.38, 1.88]) + EquivDiameter (1.19 [-0.54, 2.92]) + Extent (0.74 [0.09, 1.38]) + PrincipalAxisLength1 (-0.12 [-0.50, 0.27]) + PrincipalAxisLength2 (-0.17 [-0.57, 0.23]) + **PrincipalAxisLength3 (-0.12 [-0.37, 0.14])** + ConvexVolume (-1.82 [-3.78, 0.13]) + Solidity (-0.77 [-1.74, 0.19]) + SurfaceArea (-0.43 [-0.95, 0.09]) + age (0.4 [-0.07, 0.88]) + gender (0.22 [-0.17, 0.61]) + weight (0.28 [-0.1, 0.65]) + **BMI (-0.41 [-0.93, 0.11])** + **HBA1$_C$ (0.34 [0.10, 0.59])** + **years.of.diabetes (-0.19 [-0.36, -0.01])** + diagnosisT2D (0.06 [-0.33, 0.45]) + diagnosispreDM (0.07 [-0.18, 0.31])	0.664
		acute:MAX ~ BP_{ND} (0.01 [-0.04, 0.06], freq=0.55) + Centroid1 (0.00 [-0.06, 0.06], freq=0.6) + Centroid3 (-0.02 [-0.08, 0.03], freq=0.55) + BoundingBox1 (0.05 [-0.01, 0.11], freq=0.58) + BoundingBox3 (0.06 [-0.01, 0.13], freq=0.61) + PrincipalAxisLength3 (-0.09 [-0.15, -0.03], freq=0.63) + BMI (0.02 [-0.03, 0.07], freq=0.52) + HBA1$_C$ (0.21 [0.13, 0.28], freq=0.93) + years.of.diabetes (-0.1 [-0.17, -0.02], freq=0.63)	0.302
	Head	acute:MAX ~ **BP_{ND} (-0.01 [-0.22, 0.2])** + Volume (-1.05 [-4.7, 2.59]) + Centroid1 (-0.04 [-0.23, 0.15]) + **Centroid2 (-0.01 [-0.26, 0.25])** + **Centroid3 (0.02 [-0.28, 0.31])** + BoundingBox1 (0.42 [-0.64, 1.48]) + BoundingBox2 (0.51 [-0.9, 1.93]) + BoundingBox3 (0.45 [-0.75, 1.64]) + BoundingBoxVolume (-1.7 [-5.27, 1.87]) + EquivDiameter (-0.03 [-2.82, 2.75]) + Extent (-0.47 [-1.85, 0.91]) + PrincipalAxisLength1 (0.08 [-0.52,	1.411

		0.69]) + **PrincipalAxisLength2 (0.21 [-0.5, 0.91])** + **PrincipalAxisLength3 (-0.05 [-0.7, 0.6])** + ConvexVolume (1.6 [-2.5, 5.7]) + Solidity (0.54 [-0.97, 2.05]) + SurfaceArea (-0.08 [-0.71, 0.55]) + age (0.08 [-0.31, 0.46]) + gender (-0.12 [-0.66, 0.42]) + weight (0.04 [-0.36, 0.44]) + BMI (0 [-0.43, 0.44]) + **HBA1$_C$ (0.16 [-0.34, 0.67])** + **years.of.diabetes (-0.13 [-0.37, 0.12])** + diagnosisT2D (0.02 [-0.82, 0.85]) + diagnosispreDM (-0.15 [-0.67, 0.36])	
		acute:MAX ~ BP_{ND} (0 [-0.05, 0.06], freq=0.54) + Centroid2 (0.03 [-0.03, 0.09], freq=0.54) + Centroid3 (-0.03 [-0.08, 0.03], freq=0.56) + PrincipalAxisLength2 (0.01 [-0.06, 0.07], freq=0.61) + PrincipalAxisLength3 (-0.06 [-0.11, 0.00], freq=0.65) + HBA1$_C$ (0.22 [0.13, 0.3], freq=0.92) + years.of.diabetes (-0.10 [-0.17, -0.02], freq=0.60)	0.309
	Body	acute:MAX ~ **BP_{ND} (0.00 [-0.09, 0.09])** + Volume (-0.30 [-1.08, 0.49]) + **Centroid1 (0.13 [-0.01, 0.26])** + **Centroid2 (-0.07 [-0.20, 0.05])** + **Centroid3 (-0.01 [-0.08, 0.06])** + BoundingBox1 (-0.25 [-1.3, 0.8]) + **BoundingBox2 (-0.21 [-1.21, 0.79])** + **BoundingBox3 (-0.06 [-0.94, 0.82])** + BoundingBoxVolume (0.30 [-0.69, 1.30]) + EquivDiameter (0.30 [-1.02, 1.63]) + **Extent (0.02 [-0.63, 0.66])** + PrincipalAxisLength1 (0.29 [-0.3, 0.88]) + **PrincipalAxisLength2 (0.03 [-0.25, 0.30])** + **PrincipalAxisLength3 (0.15 [-0.19, 0.50])** + ConvexVolume (-0.27 [-1.40, 0.86]) + **Solidity (-0.08 [-0.41, 0.25])** + SurfaceArea (-0.12 [-0.26, 0.02]) + **age (-0.03 [-0.16, 0.1])** + gender (0.06 [-0.17, 0.28]) + weight (0.03 [-0.10, 0.16]) + **BMI (-0.02 [-0.14, 0.11])** + **HBA1$_C$ (0.17 [-0.04, 0.37])** + **years.of.diabetes (-0.14 [-0.32, 0.05])** + diagnosisT2D (0.15 [-0.22, 0.51]) + **diagnosispreDM (0.21 [-0.04, 0.46])**	0.445
		acute:MAX ~ BP_{ND} (0.01 [-0.05, 0.06], freq=0.67) + Centroid1 (0.07 [0.01, 0.12], freq=0.72) + Centroid2 (0.01 [-0.04, 0.06], freq=0.64) + Centroid3 (-0.01 [-0.06, 0.04], freq=0.66) + BoundingBox2 (-0.06 [-0.13, 0.02], freq=0.64) + BoundingBox3 (0.00 [-0.13, 0.14], freq=0.71) + Extent (-0.05 [-0.2, 0.09], freq=0.54) + PrincipalAxisLength2 (-0.09 [-0.16, -0.02], freq=0.75) + PrincipalAxisLength3 (0.04 [-0.06, 0.14], freq=0.51) + Solidity (-0.01 [-0.09, 0.07], freq=0.62) + age (0.01 [-0.06, 0.07], freq=0.54) + weight (0.06 [-0.02, 0.13], freq=0.60) + BMI (-0.05 [-0.13, 0.02], freq=0.56) + HBA1$_C$ (0.27 [0.2, 0.35], freq=0.97) + years.of.diabetes (-0.14 [-0.21, -0.07], freq=0.73) + diagnosispreDM (0.19 [0.05, 0.32], freq=0.56)	0.306
	Tail	acute:MAX ~ **BP_{ND} (-0.01 [-0.17, 0.15])** + Volume (0.63 [-0.64, 1.90]) + **Centroid1 (0.02 [-0.21, 0.25])** + Centroid2 (0.06 [-0.23, 0.35]) + Centroid3 (-0.02 [-0.28, 0.25]) + BoundingBox1 (0.6 [-0.14, 1.34]) + BoundingBox2 (0.53 [-0.16, 1.22]) + BoundingBox3 (0.63 [-0.26, 1.52]) + BoundingBoxVolume (0.09 [-1.67, 1.85]) + EquivDiameter (-0.32 [-1.63, 0.99]) + Extent (0.56 [-0.53, 1.64]) + PrincipalAxisLength1 (-0.25 [-1.03, 0.53]) + **PrincipalAxisLength2 (-0.21 [-0.65, 0.22])** + PrincipalAxisLength3 (-0.22 [-0.82, 0.37]) + ConvexVolume (-1.15 [-3.81, 1.52]) + Solidity (-0.44 [-1.43, 0.55]) + SurfaceArea (-0.05 [-0.24, 0.14]) + **age (0.03 [-0.12, 0.17])** + gender (-0.01 [-0.38, 0.35]) + weight (0.05 [-0.12, 0.23]) + BMI (-0.02 [-0.18, 0.14]) + **HBA1$_C$ (0.34 [0.07, 0.62])** + **years.of.diabetes (-0.12 [-0.27, 0.04])** + diagnosisT2D (-0.19 [-0.69, 0.31]) + diagnosispreDM (-0.02 [-0.39, 0.34])	2.657
		acute:MAX ~ BP_{ND} (0.02 [-0.04, 0.08], freq=0.51) + Centroid1 (0.05 [-0.01, 0.10], freq=0.57) + PrincipalAxisLength2 (-0.04 [-0.09, 0.02], freq=0.57) + age (0.05 [-0.01, 0.11], freq=0.5) + HBA1$_C$ (0.24 [0.17, 0.32], freq=0.95) + years.of.diabetes (-0.11 [-0.18, -0.04], freq=0.59)	0.276

BP_{ND} x Volume	Whole	acute:MAX ~ ***BP_{ND}* x Volume (-0.01 [-0.10, 0.09])** + Volume (-0.10 [-0.72, 0.52]) + **Centroid1 (0.05 [-0.03, 0.12])** + **Centroid2 (0.04 [-0.05, 0.14])** + **Centroid3 (0.04 [-0.04, 0.12])** + **BoundingBox1 (0.28 [-0.11, 0.67])** + BoundingBox2 (0.15 [-0.35, 0.66]) + **BoundingBox3 (0.16 [-0.21, 0.52])** + BoundingBoxVolume (0.25 [-0.43, 0.92]) + EquivDiameter (0.28 [-0.65, 1.22]) + Extent (0.35 [0.04, 0.66]) + **PrincipalAxisLength1 (0.01 [-0.23, 0.24])** + PrincipalAxisLength2 (0.08 [-0.10, 0.26]) + **PrincipalAxisLength3 (0.02 [-0.12, 0.15])** + ConvexVolume (-0.59 [-1.37, 0.19]) + Solidity (-0.21 [-0.67, 0.25]) + **SurfaceArea (-0.19 [-0.48, 0.11])** + **age (0.06 [-0.04, 0.16])** + gender (0.04 [-0.16, 0.24]) + weight (0.02 [-0.12, 0.15]) + **BMI (-0.04 [-0.18, 0.11])** + **HBA1$_C$ (0.24 [0.12, 0.36])** + **years.of.diabetes (-0.10 [-0.19, 0])** + diagnosisT2D (-0.05 [-0.33, 0.23]) + diagnosispreDM (0.06 [-0.14, 0.25])	0.521
		acute:MAX ~ *BP_{ND}* x Volume (0.01 [-0.05, 0.07], freq=0.57) + Centroid1 (0.03 [-0.03, 0.08], freq=0.78) + Centroid2 (0.01 [-0.05, 0.07], freq=0.59) + Centroid3 (-0.02 [-0.08, 0.04], freq=0.58) + BoundingBox1 (0.04 [-0.06, 0.15], freq=0.57) + BoundingBox3 (0.06 [0, 0.12], freq=0.67) + PrincipalAxisLength1 (0.05 [-0.06, 0.17], freq=0.63) + PrincipalAxisLength2 (0.02 [-0.06, 0.10], freq=0.54) + PrincipalAxisLength3 (-0.06 [-0.14, 0.03], freq=0.72) + SurfaceArea (-0.08 [-0.2, 0.04], freq=0.51) + age (0.04 [-0.01, 0.10], freq=0.71) + BMI (0.01 [-0.04, 0.06], freq=0.52) + HBA1$_C$ (0.22 [0.15, 0.29], freq=0.98) + years.of.diabetes (-0.12 [-0.20, -0.05], freq=0.69)	0.349
	Head	acute:MAX ~ *BP_{ND}* x Volume (-0.03 [-0.15, 0.09]) + Volume (-0.64 [-2.16, 0.89]) + Centroid1 (-0.05 [-0.17, 0.07]) + Centroid2 (-0.06 [-0.22, 0.10]) + Centroid3 (0.07 [-0.07, 0.21]) + BoundingBox1 (0.5 [-0.27, 1.27]) + BoundingBox2 (0.55 [-0.41, 1.51]) + BoundingBox3 (0.4 [-0.32, 1.12]) + BoundingBoxVolume (-1.50 [-3.38, 0.38]) + EquivDiameter (-0.18 [-1.66, 1.30]) + Extent (-0.33 [-0.85, 0.2]) + **PrincipalAxisLength1 (0.05 [-0.24, 0.33])** + **PrincipalAxisLength2 (0.20 [-0.20, 0.60])** + **PrincipalAxisLength3 (-0.03 [-0.48, 0.41])** + ConvexVolume (1.25 [-0.65, 3.16]) + Solidity (0.46 [-0.20, 1.11]) + SurfaceArea (-0.18 [-0.57, 0.20]) + **age (0.15 [0.00, 0.30])** + gender (-0.01 [-0.25, 0.22]) + weight (0.07 [-0.15, 0.29]) + BMI (-0.03 [-0.25, 0.19]) + **HBA1$_C$ (0.16 [-0.02, 0.35])** + **years.of.diabetes (-0.12 [-0.25, 0.00])** + diagnosisT2D (0.06 [-0.33, 0.46]) + diagnosispreDM (-0.17 [-0.56, 0.22])	0.684
		acute:MAX ~ PrincipalAxisLength1 (-0.02 [-0.07, 0.03], freq=0.56) + PrincipalAxisLength2 (0.04 [-0.02, 0.09], freq=0.59) + PrincipalAxisLength3 (-0.04 [-0.09, 0.01], freq=0.58) + age (0.05 [0.00, 0.10], freq=0.64) + HBA1$_C$ (0.2 [0.12, 0.27], freq=0.96) + years.of.diabetes (-0.11 [-0.18, -0.04], freq=0.60)	0.315
	Body	acute:MAX ~ ***BP_{ND}* x Volume (0.03 [-0.08, 0.13])** + Volume (-0.39 [-1.05, 0.27]) + **Centroid1 (0.04 [-0.06, 0.14])** + **Centroid2 (-0.05 [-0.12, 0.02])** + **Centroid3 (0.05 [-0.01, 0.11])** + **BoundingBox1 (-0.47 [-1.40, 0.45])** + BoundingBox2 (-0.48 [-1.34, 0.39]) + **BoundingBox3 (-0.32 [-1.08, 0.44])** + BoundingBoxVolume (0.3 [-0.43, 1.03]) + EquivDiameter (0.50 [-0.80, 1.80]) + **Extent (-0.29 [-0.85, 0.28])** + PrincipalAxisLength1 (0.43 [0, 0.86]) + **PrincipalAxisLength2 (0.08 [-0.08, 0.24])** + PrincipalAxisLength3 (0.24 [0.00, 0.47]) + ConvexVolume (-0.02 [-1.03, 0.98]) + **Solidity (0.08 [-0.2, 0.36])** + SurfaceArea (-0.11 [-0.25, 0.03]) + **age (0.09 [-0.02, 0.19])** + gender (0.19 [0, 0.38]) + weight (0.01 [-0.09, 0.11]) + **BMI (0 [-0.09, 0.09])** +	0.434

		**HBA1C (0.22 [0.09, 0.36])** + **years.of.diabetes (-0.2 [-0.33, -0.07])** + diagnosisT2D (0.24 [-0.03, 0.51]) + diagnosispreDM (0.08 [-0.13, 0.30])	
		acute:MAX ~ *BP*ND x Volume (0.00 [-0.07, 0.06], freq=0.58) + Centroid1 (0.01 [-0.05, 0.08], freq=0.69) + Centroid2 (-0.02 [-0.07, 0.04], freq=0.64) + Centroid3 (0.04 [-0.03, 0.1], freq=0.65) + BoundingBox1 (0.08 [0, 0.15], freq=0.66) + BoundingBox3 (0.03 [-0.04, 0.10], freq=0.61) + Extent (-0.02 [-0.11, 0.08], freq=0.56) + PrincipalAxisLength2 (-0.06 [-0.12, -0.01], freq=0.75) + Solidity (0.03 [-0.06, 0.11], freq=0.58) + age (0.07 [0, 0.15], freq=0.71) + weight (0.03 [-0.06, 0.13], freq=0.54) + BMI (-0.03 [-0.11, 0.06], freq=0.54) + HBA1C (0.24 [0.17, 0.31], freq=0.98) + years.of.diabetes (-0.11 [-0.20, -0.03], freq=0.70)	0.373
	Tail	acute:MAX ~ ***BP*ND x Volume (-0.04 [-0.16, 0.08])** + Volume (0.14 [-0.59, 0.87]) + **Centroid1 (0.02 [-0.08, 0.12])** + **Centroid2 (0.07 [-0.05, 0.18])** + **Centroid3 (0 [-0.11, 0.11])** + BoundingBox1 (0.45 [-0.07, 0.97]) + BoundingBox2 (0.45 [-0.07, 0.96]) + BoundingBox3 (0.27 [-0.22, 0.77]) + BoundingBoxVolume (-0.6 [-1.46, 0.26]) + EquivDiameter (-0.62 [-1.59, 0.35]) + Extent (0.04 [-0.4, 0.49]) + PrincipalAxisLength1 (-0.07 [-0.44, 0.29]) + **PrincipalAxisLength2 (-0.02 [-0.27, 0.24])** + PrincipalAxisLength3 (0.15 [-0.22, 0.52]) + ConvexVolume (0.26 [-0.90, 1.42]) + Solidity (0.21 [-0.2, 0.63]) + SurfaceArea (-0.04 [-0.19, 0.1]) + **age (0.08 [-0.03, 0.18])** + gender (-0.02 [-0.23, 0.2]) + weight (0.06 [-0.06, 0.17]) + BMI (-0.05 [-0.16, 0.06]) + **HBA1C (0.21 [0.07, 0.35])** + **years.of.diabetes (-0.13 [-0.23, -0.02])** + diagnosisT2D (0.13 [-0.12, 0.38]) + diagnosispreDM (0.14 [-0.08, 0.36])	0.565
		acute:MAX ~ *BP*ND x Volume (0 [-0.06, 0.06], freq=0.53) + Centroid1 (0.05 [-0.01, 0.1], freq=0.72) + Centroid2 (0.01 [-0.06, 0.07], freq=0.61) + Centroid3 (0.02 [-0.04, 0.08], freq=0.53) + PrincipalAxisLength2 (-0.02 [-0.08, 0.03], freq=0.61) + age (0.05 [-0.01, 0.11], freq=0.69) + HBA1C (0.21 [0.13, 0.28], freq=0.97) + years.of.diabetes (-0.10 [-0.18, -0.03], freq=0.63)	0.356
